# Machine Learning Methodologies Applied to Magnetocaloric Perovskites Discovery

**DOI:** 10.1021/acs.jcim.4c01944

**Published:** 2025-02-06

**Authors:** Luis E. Castro-Anaya, Eduardo Marese, Jaime A. Lozano, Guilherme F. Peixer, Jader R. Barbosa, Sergio Yesid Gómez González

**Affiliations:** † Laboratory of Mass Transfer and Numerical Simulation of Chemical Systems, Department of Chemical Engineering and Food Engineering, Federal University of Santa Catarina (UFSC), Florianópolis, Santa Catarina 88040-900, Brazil; ‡ POLO - Research Laboratories for Emerging Technologies in Cooling and Thermophysics, Department of Mechanical Engineering, Federal University of Santa Catarina (UFSC), Florianópolis, Santa Catarina 88040-900, Brazil

## Abstract

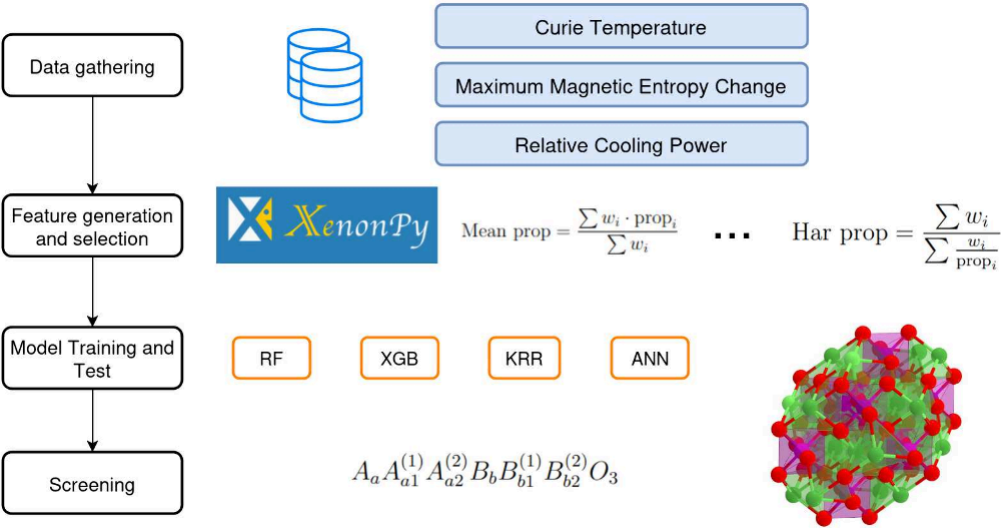

Traditionally, designing novel materials involves exploring new compositions guided by insights from previous work, relying on a trial-and-error approach, where continuous synthesis and characterization proceed until the properties meet the improvements. This method is inefficient due to the challenges of exploring vast chemical spaces. In this study, a machine-learning-based methodology is developed to assist the design from available data in the literature, allowing us to test in silico more than 1.2 million compositions. Two databases with 1227 inputs were created from published studies. Four machine learning (ML) models were trained over the feature sets using 517 compositional features (generated from 58 atomic properties) to predict magnetocaloric properties of perovskites: Curie temperature (*T*_C_), magnetic entropy change (ME), and relative cooling power (RCP). The best model-feature combinations were used to explore the chemical space of lanthanum, praseodymium, and neodymium manganites, identifying composition trends for different temperature applications, including room temperature refrigeration, where the most suitable combinations of doping elements were highlighted. The study offers valuable guidelines for future research insights on magnetocaloric materials, and the methodology can be transferred to other perovskite related material areas, such as catalysts and solar cell materials.

## Introduction

In recent years, increasing interest in magnetic refrigeration materials has fueled extensive research into the magnetocaloric effect (MCE),^1^ a phenomenon observed in specific magnetic materials and is characterized by a temperature change when the materials are subjected to a variation in the external magnetic field applied to them.^2^

The effect occurs due to the reordering of magnetic spins within the material. The intensity of this phenomenon is commonly described by a curve of temperature against magnetic entropy change at a specified magnetic field.^3^ This curve starts from a low value and increases, reaching a maximum value near the transition between two magnetic states, such as the Curie temperature, where the transition from the ferromagnetic to paramagnetic state occurs. After the peak is reached, the curve decreases back. The peak of the curve is known as the maximum magnetic entropy change, and this metric along with Curie temperature are typically used to characterize the MCE in terms of potential refrigeration applications and intensity. Additionally, a figure of merit known as the relative cooling power considers the width of the peak, as illustrated by eq 1.
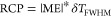
1where RCP is relative cooling power, ME is magnetic entropy change, and *δT*_FWHM_ is the temperature range over which the entropy change is the half of ME.

As a promising alternative to conventional cooling systems, the magnetic refrigeration field demands a meticulous selection of materials to ensure efficient, reliable, and sustainable cooling solutions, considering the complexity of thermodynamic processes and specific temperature requirements across different applications, ranging from room-temperature^4−6^ to cryogenic^7^ systems. Due to the reversibility of the MCE and work recovery during the demagnetization process, magnetocaloric systems have the potential to operate at high thermodynamic efficiencies.^8^ Moreover, the application of solid-state refrigerants prevents the leakage of environmentally harmful substances, increasing the interest in the technology for sustainability factors. Hence, research on magnetic refrigeration is attracting increasing attention and application prospects under a wide range of operation temperatures.^9^

Perovskites, distinguished by their general formula ABO_3_, have garnered substantial interest in the quest for novel magnetocaloric materials, owing to their exceptional crystalline structure and adaptable magnetic properties. The diverse compositions and adjustable properties of perovskites offer an array of options for tailoring magnetocaloric responses, enabling the design of efficient cooling devices adaptable to various temperature ranges and chemical environments from the material’s point of view. Perovskites also have the potential to lower costs and facilitate fabrication features; for instance, manganites possess the mentioned advantages and are more economical and accessible to prepare than classic magnetocaloric materials such as intermetallic alloys.^10,11^

Exploration of this family of materials is commonly performed through experiments using a trial-and-error approach;^9,12^ however, recently, computational studies employing Monte Carlo molecular simulations and first-principles calculations have also been carried out.^13,14^ The experimental scenario is limited due to its time-consuming and resource-demanding nature. In the computational simulation scenario, time remains a constraint since those simulations are computationally intensive, and the accuracy of the computations relies on the ability of the Hamiltonian to reproduce the atomistic physical reality.^15,16^ An alternative to these two scenarios is to use data-driven methodologies such as machine learning approaches.

Machine learning (ML) methodologies rely on a limited but significant amount of experimentally obtained or simulated data, together with the extraction of features from the collected data to enable the application of algorithms, ensuring that the most relevant attributes are considered during model development, for training and optimization of ML models.^17^ Once trained, these models can efficiently explore a vast chemical space, requiring minimal resources. Remarkably, these scenarios can be complemented, as in the case of active experimentation, using iterative refinement of ML models.^18^

Data-driven methodologies have been used to explore material families such as, e.g., Heusler intermetallic compounds.^19−21^ Notable in this field is the work of Ucar et al.,^22^ who studied the maximum magnetic entropy change (ME) in several families of compounds, including some manganites, finding that the distinct features of each family explain the variations in the MCE. Their research highlights the importance of studying each family separately to achieve accurate results. Before the study of Ucar et al., only two ML investigations focused on perovskites: Zhang and Xu^23^ investigated the Curie temperature (*T*_C_) of 94 manganites employing the crystal lattice constants as features and a Gaussian Process Regressor (GPR) as a ML model. The authors obtained a coefficient of determination of 0.7, which is remarkable considering the feature set used but not very significant, given the amount of data and the absence of a test set. The other work in this regard comes from Zhai et al.,^24^ who assessed 3 ML methods over 47 perovskites to predict the *T*_C_. Despite having an excellent selection of structural and compositional parameters, they obtained a moderate coefficient of determination of low significance due to the absence of a testing stage equal to 0.85.

As previously mentioned, the amount of data available or employed has limited the development of robust models. Moreover, the unique MCE-related property explored was *T*_C_. Fortunately, this picture has changed, as the available number of experimental data has increased to the extent that there are incentives for ML study in this family of materials.^25^ Additionally, the conducted reviews^12,25^ have pointed out the importance of crystallite size in describing MCE in perovskites, which has not been taken into account by the previous studies. Crystallite size corresponds to a region within a crystalline material with a uniform crystal lattice orientation.^26^ This morphological parameter has a direct effect on material properties. Its control is widely used to optimize materials for applications such as semiconductors^27^ and magnetocaloric materials.^28^ Hence, an opportunity arises to evaluate this parameter as a morphological characteristic to better predict the thermomagnetic properties of magnetocaloric materials better.

In response to those needs, this work critically studies ML models, data collection, and feature selection procedures for predicting magnetocaloric properties (*T*_C_, ME, and RCP) on perovskite materials. Four ML models are evaluated over different subsets of compositional features. A morphological descriptor is also employed, and their effect on the above-mentioned properties is assessed and discussed. Finally, the best ML models are used to explore the chemical subspace, and several insights are outlined for future studies of this family of materials.

## Methods

**Figure 1:**
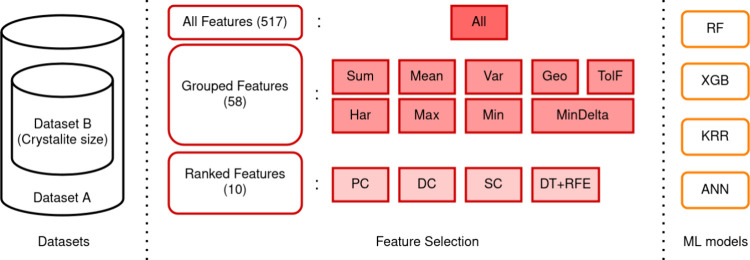
Scheme showing the steps of the methods of this work: The left part presents the datasets used without crystallite size (Dataset A) and with crystallite size (Dataset B). The middle part shows the classification and nomenclature of the feature sets used. The right part shows the ML models employed.

Herein, a systematic approach is presented to collect data on perovskite compositions and the three magnetocaloric properties: *T*_C_, ME, and RCP. Detailed insights into the data preprocessing methods and feature engineering techniques are also provided. Furthermore, we describe the ML models and used parameters, emphasizing their applicability in predicting the magnetocaloric properties of interest. Figure 1 depicts the main variations in the dataset, feature set, and ML model employed.

### Dataset

The data employed in this work were collected by manually extracting the three magnetocaloric properties of interest found in graphics and tables from 146 references (note: the list of article Digital Object Identifiers (DOIs) is available in the GitHub open repository (https://github.com/luis-chemeng/MCML)), generating a dataset for each property. The dataset contains the chemical formula of perovskites, the value of the property, and the crystallite size (*D*) reported along with the XRD measurements. The magnetic field applied is also reported in the cases of ME and RCP. After ensuring the chemical formulas’ data quality and validity, a separate database was produced for each magnetocaloric property. In each dataset, the records were filtered to consider just single perovskites with a stoichiometric composition of oxygen. The resulting datasets contained missing values on the crystallite size column; we explored two alternatives, as ML models can not be trained over features with empty values. In the first one, the feature crystallite size was disregarded, preserving the total number of samples of the dataset, which led to the denominated Dataset A. The second strategy consisted of using only the data samples with the crystallite size information, privileging the information provided by the crystallite size at the expense of the data quantity, leading to the denominated Dataset B, as presented in Figure 1.

As a result of not considering the crystallite size in Dataset A, some records of this dataset contained several values of the property of interest for the same chemical composition. This situation increases the error in the ML models and decreases their performance in generalizing the data. To overcome this situation, we eliminate the repeated records and leave only the record corresponding to the median of the property values.

#### Reliability of the Collected Data

Since ML models depend on the quantity and quality of data, it is worth making some notes regarding the reliability of the experimental data gathered: (i) It is well-known that the magnetocaloric characterization of materials is subject to experimental errors. In particular, the references from which the data were extracted measured the magnetic entropy change indirectly using magnetization curves and the Maxwell relation. In this context, Pecharsky and Gschneidner established that the uncertainty is as low as 10% relative deviation.^29^ This partially explains the dispersion observed in the magnetocaloric properties measured for materials with the same composition. (ii) Manganites are well-known to undergo second-order magnetic phase transitions.^10^ This is confirmed in most of the reference works through the Arrott plot slope analysis. This aspect increases our confidence in the reported values, as second-order phase transitions are less prone to hysteresis behavior that could otherwise hinder the accurate measurement of magnetocaloric phenomena. (iii) Manufacturing conditions can lead to inhomogeneities that are not always confirmed by experimental techniques, such as energy-dispersive X-ray spectroscopy (EDX). Additionally, different morphological aspects, such as crystallite size and anisotropy, were not always reported or verified.

### Feature Generation and Selection

Through the Python module XenonPy,^30^ a table containing 58 atomic properties for every element of the periodic table was obtained. Each property is described in the Supporting Information in Table S1. XenonPy also offers 7 combinational formulas to obtain compositional descriptors from the atomic properties of the constitutive elements of the compound, namely, Weighted Average, Weighted Sum, Weighted Variance, Geometric Mean, Harmonic Mean, Maximum Aggregation, and Minimum Aggregation. This resulted in a vector comprising 406 features. The mathematical expressions of these combinational formulas are in the Supporting Information in Equations S1 to S7. We include two new combinatorial formulas. The first one is the minimum difference between sites (eq S8 of Supporting Information), which is the minimum value between the difference of atomic property of site A or B with the oxygen atomic property, which accounted for 58 additional descriptors. This combination was inspired by the high dependence shown between this descriptor and the bandgap energy of perovskites reported by Körbel et al.^31^ and subsequently used by Gladkikh et al.^32^ The second one is the tolerance factor-like descriptor (eq S9 of Supporting Information), which has a formula analogous to the Goldschmidt tolerance factor, but instead of using the ionic radii of the A, B, and oxygen sites, we used all 58 atomic properties available in XenonPy. As some of the atomic properties are zero for B site and oxygen, the calculation of this formula was impossible for 5 of the atomic properties, resulting in 53 descriptors. This combinatorial formula is used as Franco et al.^33^ showed that the tolerance factor is moderately correlated with *T*_C_. This resulted in a vector comprising 517 features designated as compositional descriptors. The crystallite size was used as a morphological descriptor without further refinement.

Gladkikh et al.^32^ have reported how to select features for input to a ML algorithm and how to map these features to the target variable. They perform a correlation analysis with the descriptors and the property of interest using various correlation coefficients, such as Pearson and Spearman coefficients, Distance Correlation, etc. It is concluded that the degrees of correlation between a feature and the property in question should be regarded as something other than definitive, as they can vary depending on the correlation coefficient employed.

Four schemes of feature selection were implemented over the compositional descriptors: First (i) the features were grouped according to the combination type used, giving rise to 9 groups, each one composed of 58 features, except for the tolerance factor-like features group, which had 53 features. (ii) The second approach ranked the 517 features with their correlation coefficient concerning the objective property; the best 10 performers were selected. Three kinds of correlation coefficient calculations were applied: Pearson coefficient (PC), which measures the strength and direction of the linear relationship between two continuous variables; distance correlation (DC), which captures both linear and nonlinear relationships between variables; and the Spearman coefficient (SC), which evaluates the strength and direction of the monotonic relationship between two variables. We refer to this set of descriptors as PC10, SC10, and DC10, respectively. To ensure the most unique information was captured, a decorrelation strategy was used; i.e., the features with a correlation higher than 0.9 with a higher-ranked feature were discarded. (iii) Feature selection based on decision tree regressor (DT) was employed. For this, a decision tree regression model was trained. The relevance of the features was ranked according to the recursive feature elimination (RFE) method, which is a feature selection technique that works by iterative elimination of the less relevant or negligible features (those with the lowest Gini coefficient) until a desired number of features is left, in this case, 10. This set of features was called RFE10. Finally, (iv), a set without any further feature selection containing the total of compositional descriptors, named “All”, was also evaluated.

### Machine Learning Models

The ML models were employed to model the magnetocaloric properties in conjunction with every set of features previously obtained. The data were divided for each property, with respect to a balance of 80% into a training set and 20% into a test set. The training set was further divided into 5 subsets, and using a five-round training process, 4 of the 5 subsets were used for training, and then the remaining subset was used for validation. This procedure, known as 5-fold cross-validation, was applied for a grid of hyperparameters to address the best nonoverfitting combination of hyperparameters for each model, i.e., the one that showed the minor root-mean-square error average on the validation sets. This step was done through the Bayesian optimization implemented in the Python module Hyperopt.^34^ The performance of optimized models was evaluated over the test set using three key metrics: root mean squared error (RMSE), which measures the square root of the average squared errors, given by Equation 2; mean absolute error (MAE), which measures average absolute errors, given by Equation 3; and the coefficient of determination (*R*^2^), which measures the proportion of explained variance, varying from 0 to 1, given by Equation 4.
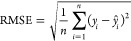
2
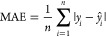
3
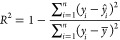
4Here *y*_*i*_ represents the observed values of the dependent variable, *ŷ*_*i*_ represents the predicted values of the dependent variable, and *y̅* represents the mean of the observed values of the dependent variable.

Four different ML models were evaluated in the present work. The first one is kernel ridge regression (KRR). This regression technique extends traditional ridge regression by leveraging the kernel technique, allowing it to handle nonlinear relationships between predictors and the target variable.^35^ KRR operates by minimizing the regularized sum of squared errors between the ground truth and the predicted values. Unlike linear regression methods, KRR employs a kernel function to implicitly map the input features into a higher-dimensional space where a linear model is fitted to capture intricate patterns in the data. This kernel-based approach enables KRR to model complex relationships flexibly while maintaining computational efficiency. The hyperparameters optimized for this model were the regularization strength, kernel type, and kernel coefficient for each kernel type. Each hyperparameter range is detailed in Table S2, in the Supporting Information.

The second model is artificial neural networks (ANN), an ML model that copies the functioning of the human brain. It comprises artificial neurons located in different layers, which process information and learn patterns according to the data provided.^36^ According to the complexity of what is being predicted, it is possible to modify the architecture of the neural network, changing the number of hidden layers and the number of neurons in each of these layers, as well as the activation function, which is a mathematical function that enables the ANN to model complex nonlinear patterns. The ANN is implemented as the multilayer perceptron regressor class available in the sklearn python module.^37^ A term accounting for the square of the neuron weights was included to avoid overfitting. This technique is known as L2 regularization. The evaluated hyperparameters are the number of layers and the number of neurons in each layer, the L2 regularization parameter, and the activation function. Each range of hyperparameter used is detailed in Table S3, in the Supporting Information. The optimizer employed is the Adam solver^38^ with an initial learning rate value of 0.01. This learning rate was implemented as adaptive, meaning that the learning rate can change during the training based on the model’s performance.

The next evaluated model is the random forest (RF), an ensemble ML technique that combines multiple decision trees to enhance predictive accuracy and reduce overfitting.^36^ It relies on generating several bootstrap samples, where data points are randomly selected from the original dataset with replacement, forming subsets with potential overlaps. Multiple decision trees are built using these samples, each considering a random subset of features at each split to introduce variability and decorrelate the trees, thus, mitigating overfitting. In the end, predictions from individual trees are either averaged for regression tasks or subjected to a majority vote for classification tasks, providing more stable and accurate predictions while minimizing the impact of outliers and noise. As in the previous models, the implementation available in the sklearn module is used.^37^ The tuned hyperparameters included the number of estimators, i.e., the number of decision trees, the depth of the trees, the minimum number of samples for each split, and the minimum number of samples in each leaf. Each range of hyperparameters used is detailed in Table S4 in the Supporting Information.

The last model to be evaluated is XGBoost (Extreme Gradient Boosting). Like the previous model, XGBoost is an ensemble ML algorithm that creates a predictive model by sequentially adding decision trees, each aimed at correcting the errors made by the preceding ones.^39^ These decision trees are constructed using a weighted dataset, where the weight is adjusted based on the errors from the previous trees. The algorithm also incorporates regularization techniques to control the overfitting. XGBoost enhances its performance by optimizing a loss function and applying a gradient descent-based approach. This method allows it to efficiently fit complex relationships within the data, making XGBoost a highly competitive algorithm for various ML tasks such as classification and regression. This model was constructed by using the XGBRegressor class in the xgboost python module. The hyperparameters optimized for this model include the number of estimators, the maximum depth of a tree, the learning rate, the subsample ratio of the training instances, and the subsample ratio of columns when constructing each tree. Each range of hyperparameters used is detailed in Table S5 in the Supporting Information.

### Chemical Space Screening

The systematic formula given by Equation 5 generated a chemical space with potential perovskites. Letters A and B correspond to the A and B sites of the single perovskite; superscripts 1 and 2 indicate the doping elements of the principal site (the one without superscripts). Element in principal site A was limited to La, Sr, and Nd as the three most common and abundant components in our database, and the concentration varies between 0.6 and 1. In the case of the B principal site, only Mn was considered, as the database is almost exclusively made of manganites. The concentration of Mn varied from 0.8 to 1. The doping sites A and B were selected from the elements in our database in sites A and B, respectively. The doping site concentration was incrementally adjusted in intervals of 0.05, leading to a set of more than 1.2 million compositions.
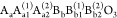
5

## Results and Discussion

### Dataset Description

**Figure 2:**
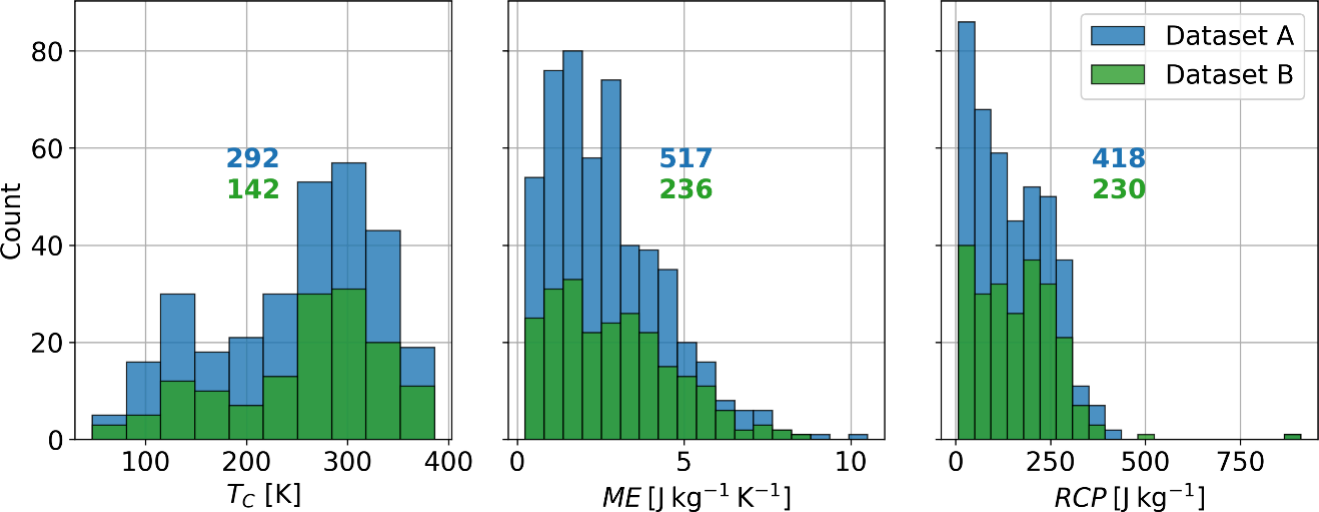
Histograms of the three magnetocaloric properties indicating the total amount of data (blue) and subset of data with the crystallite size (green). a) *T*_C_ distribution. b) ME distribution and c) RCP distribution.

The database contains 292, 517, and 418 records for *T*_C_, ME, and RCP, respectively. The distribution of each property is presented in blue in Figure 2. A second distribution, accounting just for the records with crystallite size reported, is displayed in green in Figure 2. As can be seen, the distribution is similar, while the quantity of data is about half (48.6%, 45.6%, and 55.0% for *T*_C_, ME, and RCP, respectively). The absence of this parameter in more than half of the studies consulted is somewhat surprising because, even when most of the studies have XRD characterization and analysis, the calculation of crystallite size was omitted.

**Figure 3:**
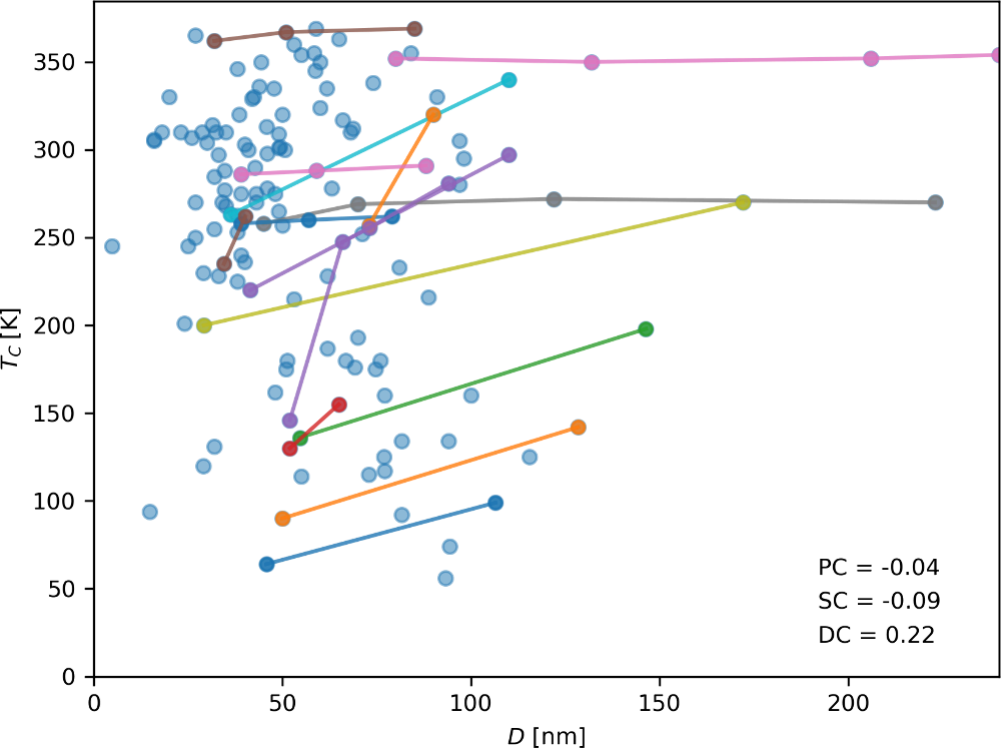
Effect of crystallite size (*D*) on *T*_C_. Pearson correlation coefficient (PC), Spearman correlation coefficient (SC), and distance correlation coefficient (DC) are displayed for all data points. The data points collected in the same study are represented by lines of different colors.

**Figure 4:**
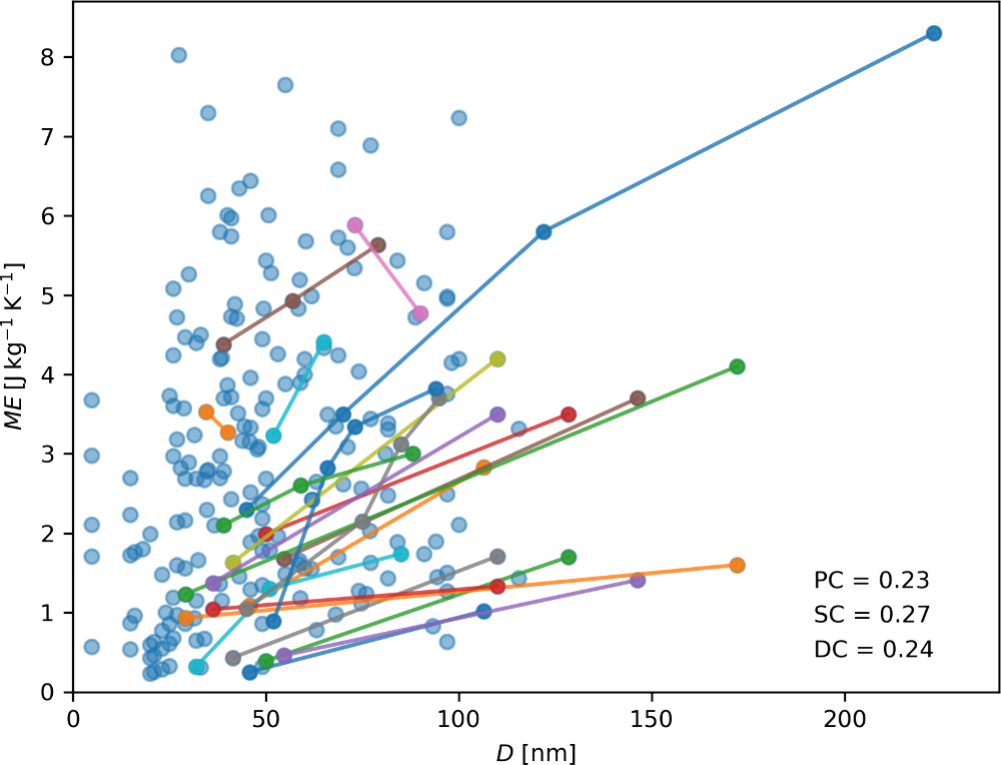
Effect of crystallite size (*D*) on ME. Pearson correlation coefficient (PC), Spearman correlation coefficient (SC), and Distance correlation coefficient (DC) are displayed for all data points. The data points collected in the same study are represented by lines of different colors.

**Figure 5:**
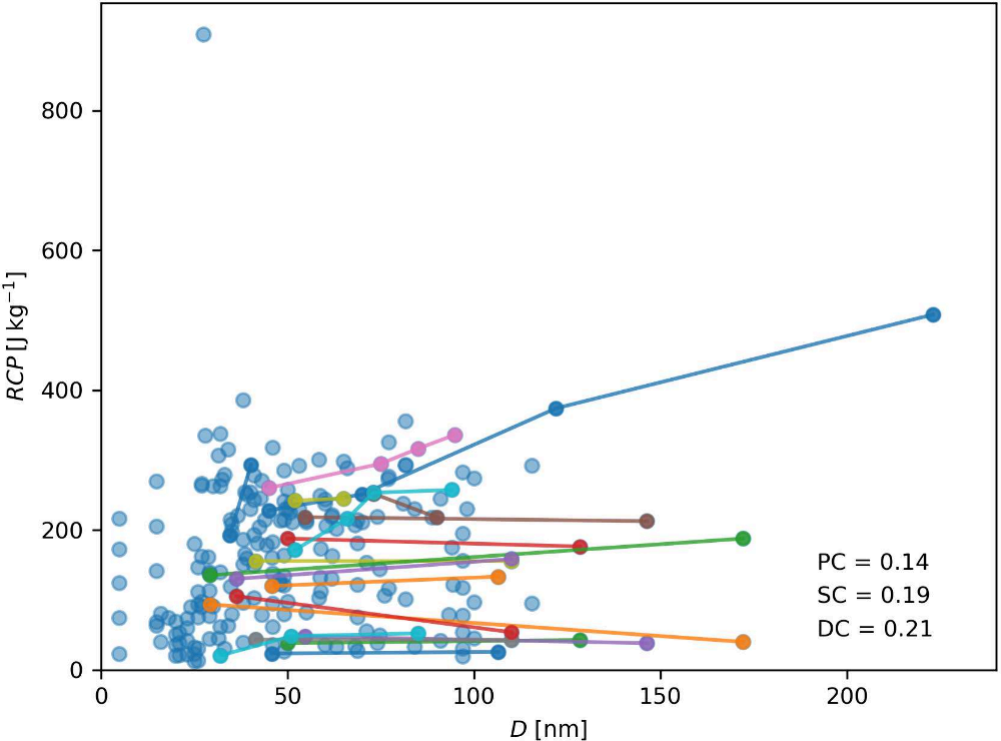
Effect of crystallite size (*D*) on RCP. Pearson correlation coefficient (PC), Spearman correlation coefficient (SC), and distance correlation coefficient (DC) are displayed for all data points. The data points collected in the same study are represented by lines of different colors.

The effect of crystallite size on the magnitude of magnetocaloric properties in studies for specific compositions has been reported previously.^40−42^ Records with reported crystallite sizes for each of the properties were plotted to evidence the effect of crystallite size through all the data collected, as seen in Figures 3–5. It should be noted that the data in Figures 4 and 5 depict data for a wide range of magnetic fields. In particular, Figure 5 displays an exceptionally high RCP value, corresponding to a magnetic field of 14 T. The values highlighted in different colors are from studies in which the synthesis conditions were varied (such as the synthesis method, sintering temperature, sintering time, or applied pressure). Therefore, perovskites with the same chemical composition but different morphology and, ultimately, different crystallite sizes were obtained. For *T*_C_, the effect of crystallite size is proportional. However, the magnitude of this proportionality varies among the different perovskites. For some of the compositions, the effect is significant, while for others, it is almost null as the size of the crystallite increases, i.e., an asymptotic behavior. The effect generally seems proportional and more intense than *T*_C_ for the ME. However, it is possible to see two studies where this effect is reversed. Something similar happens with the RCP, where although the effect also seems generally positive, there are a couple of studies with an inversely proportional effect; this is not surprising as RCP is defined in terms of ME, being directly proportional as per established Equation 1.

Regarding a general trend and the use of correlation coefficients, although a discernible trend is observable in the scatter plots, the correlation coefficient fails to confirm this relationship, especially in the case of *T*_C_, where the Pearson and Spearman coefficients depict a negative trend. For ME and RCP, the coefficients are low. However, the effect is indeed palpable. These results highlight the need for correlation coefficients in discerning meaningful trends in this material informatics problem, as they considered only the impact of one variable at a time.

### Feature Selection Comparison

We utilized the correlation coefficients described in the methods section to determine the ranked features, ranking the features based on these coefficients, and selecting the top 10 features for each coefficient. This approach allowed us to evaluate how the models perform with fewer features.

Tables 1–3 present the best 10 features for *T*_C_, ME, and RCP, as their corresponding feature selection coefficients indicate. It is worth mentioning that the features in the ranked sets based on correlation coefficient do not possess a high intercorrelation, and they are not directly comparable because of the decorrelation strategy undertaken. Despite the above, the appearance of certain types of features stands out in the ranked sets. The features ranked according to the correlation coefficients have, in general, very similar positions in the rankings. However, looking at the types of features ranked by the RFE method may differ from those for the PC, SC, and DC coefficients, highlighting the importance of carrying out this feature selection with multiple methods. In the particular case of *T*_C_, the features related to the number of unfilled electrons or valence electrons in the last atomic shells appear as the most significant feature in the four sets. Indeed, Sight et al., who studied rare-earth intermetallic compounds, found that the 4f electron count significantly influences the *T*_C_ magnitude. This atomic property was also a significant driver feature in analyzing the critical temperature of superconductive materials.^43^ In the case of ME, the property that stood out the most was the lattice constant. Oppositely, for RCP, no single elemental property emerged as prominently significant. This indicates a more complex interaction of factors influencing RCP and highlights the importance of a comprehensive analysis in understanding their behavior.

**Table 1:** Best 10 Predictors According to the Feature Selection Methods (DC, SC, PC, RFE) for *T*_C_

**Rank**	**PC**_**decorr**	**SC**_**decorr**	**DC**_**decorr**	**RFE**
**1**	Sum num_f_unfilled	TolF_electron_affinity	TolF_electron_affinity	Mean en_pauling
**2**	Mean num_f_unfilled	MinDelta_num_d_unfilled	MinDelta_num_d_unfilled	Mean num_f_unfilled
**3**	MinDelta_num_d_unfilled	TolF_evaporation_heat	Mean num_d_unfilled	Var en_allen
**4**	Mean num_d_unfilled	Min en_allen	Sum num_d_unfilled	Geo electron_affinity
**5**	Sum num_d_unfilled	Max num_d_unfilled	Sum num_f_unfilled	Geo lattice_constant
**6**	Var num_f_unfilled	TolF_heat_of_formation	Mean num_f_unfilled	Har num_valence
**7**	MinDelta_electron_affinity	Har fusion_enthalpy	TolF_num_d_unfilled	TolF_electron_affinity
**8**	Max num_d_unfilled	Sum num_f_unfilled	MinDelta_electron_affinity	MinDelta_bulk_modulus
**9**	TolF_electron_affinity	Mean num_f_unfilled	Min en_allen	MinDelta_hhi_r
**10**	Min en_allen	Mean num_d_unfilled	TolF_evaporation_heat	MinDelta_num_valence

**Table 2:** Best 10 Predictors According to the Feature Selection Methods (DC, SC, PC, RFE) for ME

**Rank**	**PC**_**decorr**	**SC**_**decorr**	**DC**_**decorr**	**RFE**
**1**	Min lattice_constant	MinDelta_heat_capacity_molar	TolF_lattice_constant	Sum electron_affinity
**2**	Max bulk_modulus	TolF_lattice_constant	MinDelta_hhi_r	Sum num_f_unfilled
**3**	MinDelta_thermal_conductivity	Max bulk_modulus	MinDelta_heat_capacity_molar	Var melting_point
**4**	MinDelta_hhi_r	MinDelta_thermal_conductivity	MinDelta_atomic_radius_rahm	Var molar_volume
**5**	TolF_lattice_constant	Min lattice_constant	MinDelta_hhi_p	Geo electron_negativity
**6**	Sum electron_affinity	Mean melting_point	MinDelta_covalent_radius_cordero	Geo num_valence
**7**	MinDelta_hhi_p	Max melting_point	Mean melting_point	Har bulk_modulus
**8**	Max melting_point	Min thermal_conductivity	Sum electron_affinity	Har lattice_constant
**9**	Mean electron_affinity	TolF_heat_of_formation	Min lattice_constant	Har sound_velocity
**10**	Mean bulk_modulus	Min atomic_radius	MinDelta_covalent_radius_slater	TolF_vdw_radius_alvarez

**Table 3:** Best 10 Predictors According to the Feature Selection Methods (DC, SC, PC, RFE) for RCP

**Rank**	**PC**_**decorr**	**SC**_**decorr**	**DC**_**decorr**	**RFE**
**1**	Sum electron_affinity	Min thermal_conductivity	MinDelta_vdw_radius_alvarez	Sum heat_capacity_molar
**2**	Mean electron_affinity	Var atomic_number	MinDelta_vdw_radius	Sum thermal_conductivity
**3**	MinDelta_vdw_radius_alvarez	Sum electron_affinity	MinDelta_atomic_radius_rahm	Mean en_allen
**4**	Mean evaporation_heat	Har heat_capacity_molar	MinDelta_num_unfilled	Mean hhi_r
**5**	Mean heat_capacity_molar	Geo heat_capacity_molar	Mean thermal_conductivity	Mean heat_capacity_molar
**6**	MinDelta_atomic_radius_rahm	Max electron_affinity	Sum thermal_conductivity	Var atomic_radius_rahm
**7**	Geo heat_capacity_molar	Geo electron_affinity	MinDelta_c6_gb	Var covalent_radius_cordero
**8**	Har heat_capacity_molar	Sum heat_capacity_molar	MinDelta_covalent_radius_pyykko	Har hhi_p
**9**	Geo electron_affinity	Mean heat_capacity_molar	MinDelta_atomic_radius	TolF_heat_capacity_mass
**10**	Sum lattice_constant	Min covalent_radius_slater	Sum electron_affinity	TolF_num_valence

### Evaluation of Machine Learning Models

**Figure 6:**
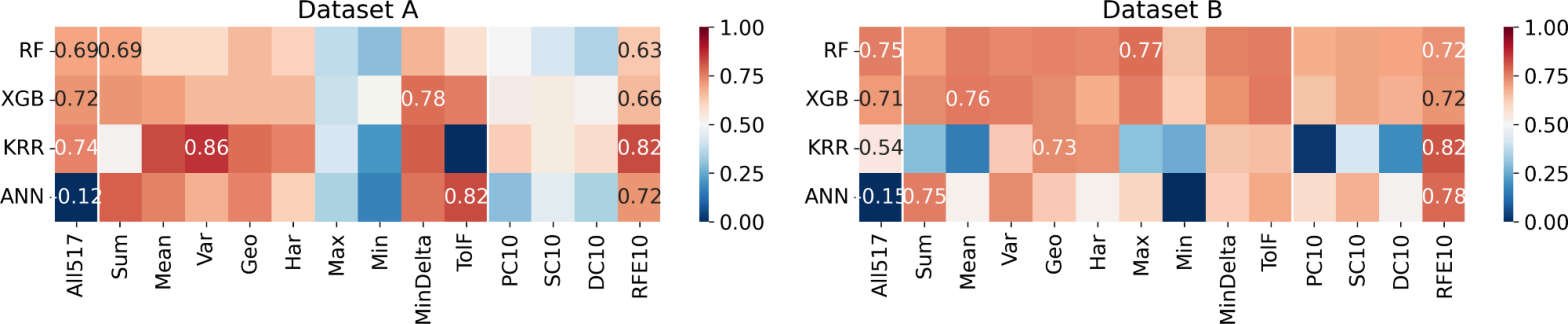
Heatmaps indicating the *R*^2^ in the prediction of Curie temperature (*T*_C_), using different combinations of ML models (*y*-labels) and feature sets (*x*-labels). The squares with numbers represent better results among the feature sets. The performance using all compositional features (All517) is always displayed.

Performance for a combination of the dataset used, optimized ML model, and feature set used was evaluated using *R*^2^ metric over the test set. Those results are depicted as heatmaps in Figures 6, 8, and 10 for *T*_C_, ME, and RCP, respectively.

The scale (color) goes from 0 (dark blue) to 1 (dark red). Thus, red tones correspond to coefficients of determination greater than 0.5, while blue tones correspond to values below this threshold. The numerical values for the best performers in each feature set category are displayed within the respective cell of the heatmap.

Although the trends for each predicted property are different and will be discussed below, we observed a general trend: using the whole 517 descriptors does not perform significantly better than using grouped feature sets or ranked feature sets. Even for some specific models, the performance was much worse. This can be explained by the fact that an unnecessarily large number of inputs bring noise to the model and reduce its ability to find general patterns.

#### Curie Temperature

The heatmap of *R*^2^ for the combination of the ML model and feature set evidence some exciting aspects: (i) for Dataset A, models trained with maximum and minimum pooling atomic properties (Max and Min descriptors, respectively) evidence lower performance than the other descriptors across the 4 ML models tested. This suggests that the descriptors obtained by these combinations contain little relevant information for describing the *T*_C_, compared with other sets of descriptors. This observation is still valid in Dataset B for the Min feature set. Meanwhile, the Max feature set performs notably well in Dataset B with the XGB and RF models; and (ii) looking at the ranked feature sets for both datasets, the RFE selection methodology was dominant. Achieving even the best *R*^2^ score among all of the feature sets for ANN and KRR models in Dataset B.

**Figure 7:**
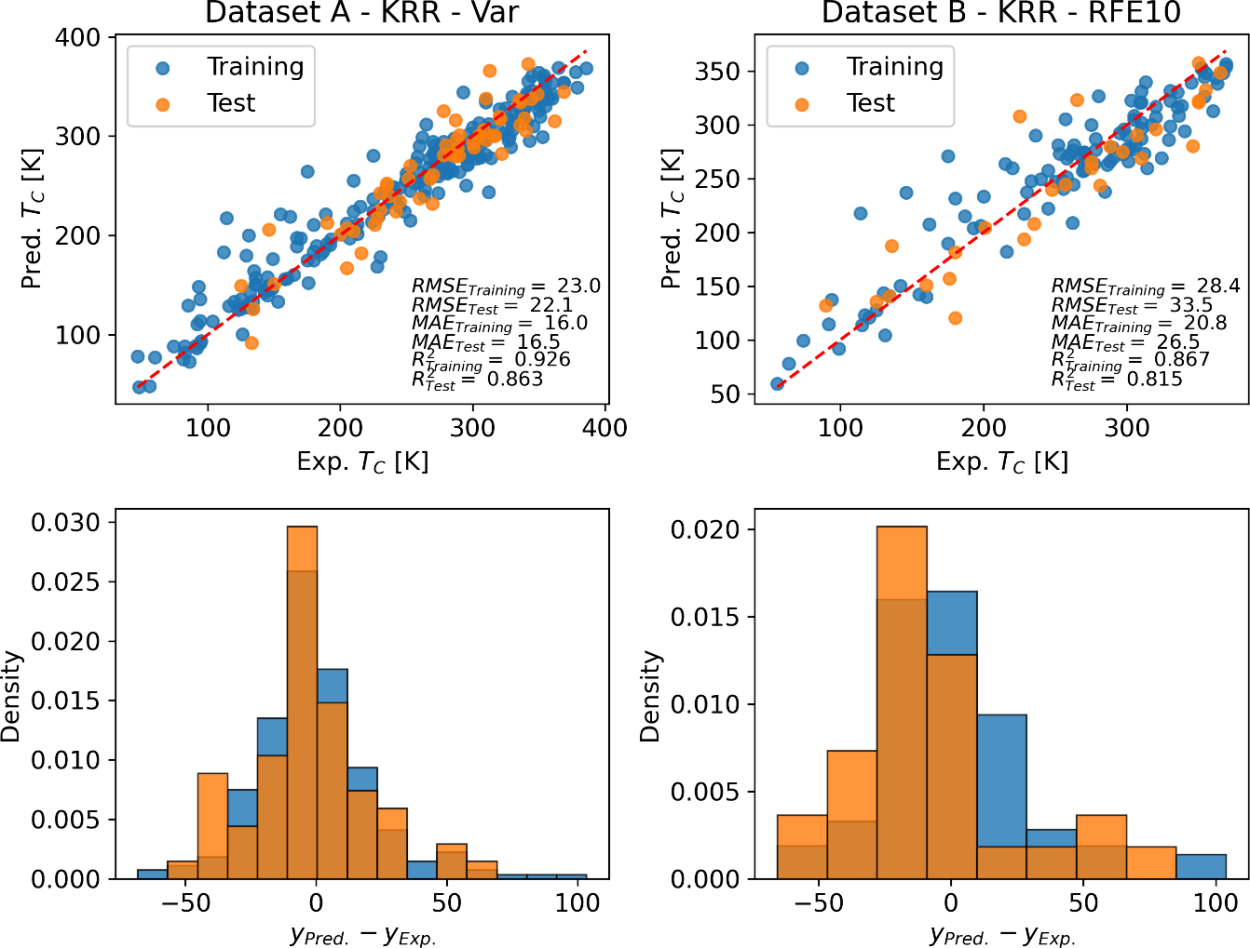
Parity plots of the best combination of ML models and feature set for experimental vs predicted *T*_C_ of magnetocaloric perovskites for training (blue) and test (orange) data. Below each parity plot is the respective normalized histogram of the residuals.

As the intended use of ML models in this work is to predict the magnetocaloric properties, Figure 7 shows the best combination of the ML model and feature set for *T*_C_ prediction along with the parity plots and performance metrics for Datasets A and B. Figure 7 also presents histograms of residuals, indicating the difference between the ground truth and the data predicted for the models. For Dataset A, the training and test histograms display the same mode, indicating consistency across the training and testing data. However, a notable distinction emerges for Dataset B: the training and test histograms showcase different modes, notably skewed toward negative values of deviations. This shift suggests a systematic underestimation of experimental values within the test set, highlighting potential discrepancies in the prediction accuracy. The best model was the kernel ridge regression (KRR), using the Dataset A and “Weighted Variance”, resulting in an RMSE of 22.1 K, MAE of 16.5 K and *R*^2^ of 0.86. Those metrics are comparable to those obtained by Zhai et al.:^24^ RMSE of 28.6 K, MAE of 21.21 K, and *R*^2^ of 0.85. However, note that the metrics from this author were over the validation set and not on the test set. Therefore, the generality of the model expressed by Zhai et al. is not assured. Our results are also comparable with the ones of Court et al.,^44^ which employed the XenonPy module to generate descriptors to predict *T*_C_ in Heusler alloys. They obtained a RMSE of 60 K and a *R*^2^ of 0.71 using a XGB model, suggesting that better results on *T*_C_ prediction can be obtained using fewer features.

Regarding crystallite size and its effect as a feature, it was found to have the lowest impact on the variation of *T*_C_. A detailed discussion of this matter is available in the Supporting Information under the section Interpretability of the ML Models.

#### Maximum Magnetic Entropy Change

**Figure 8:**
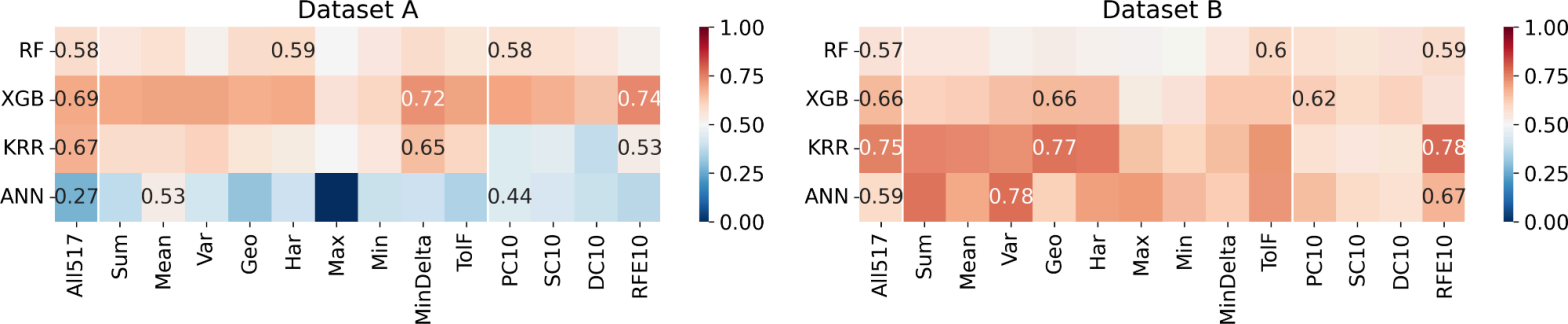
Heatmaps indicating the *R*^2^ in the prediction of maximum magnetic entropy change (ME), using different combinations of ML models (*y*-labels) and feature sets (*x*-labels). The squares with numbers in them represent results among the feature sets. The performance using all compositional features (ALL517) is always displayed.

Figure 8 indicates that for Datasets A and B, no feature set stands out inside the grouped features, contrasting with the ranked features, where the RFE10 descriptors emerge as the feature set that optimally trains most models according to the *R*^2^. As for the *T*_C_ prediction, models trained on Dataset A demonstrate that the maximum and minimum pooling atomic properties (Max and Min descriptors, respectively) exhibit inferior performance compared to those of other descriptors across all four ML models tested. Regarding Dataset A specifically, the ANN model consistently performs negatively compared to other models, regardless of the feature set employed. However, the XGB model stands out positively for this dataset, showcasing superior predictive capability.

**Figure 9:**
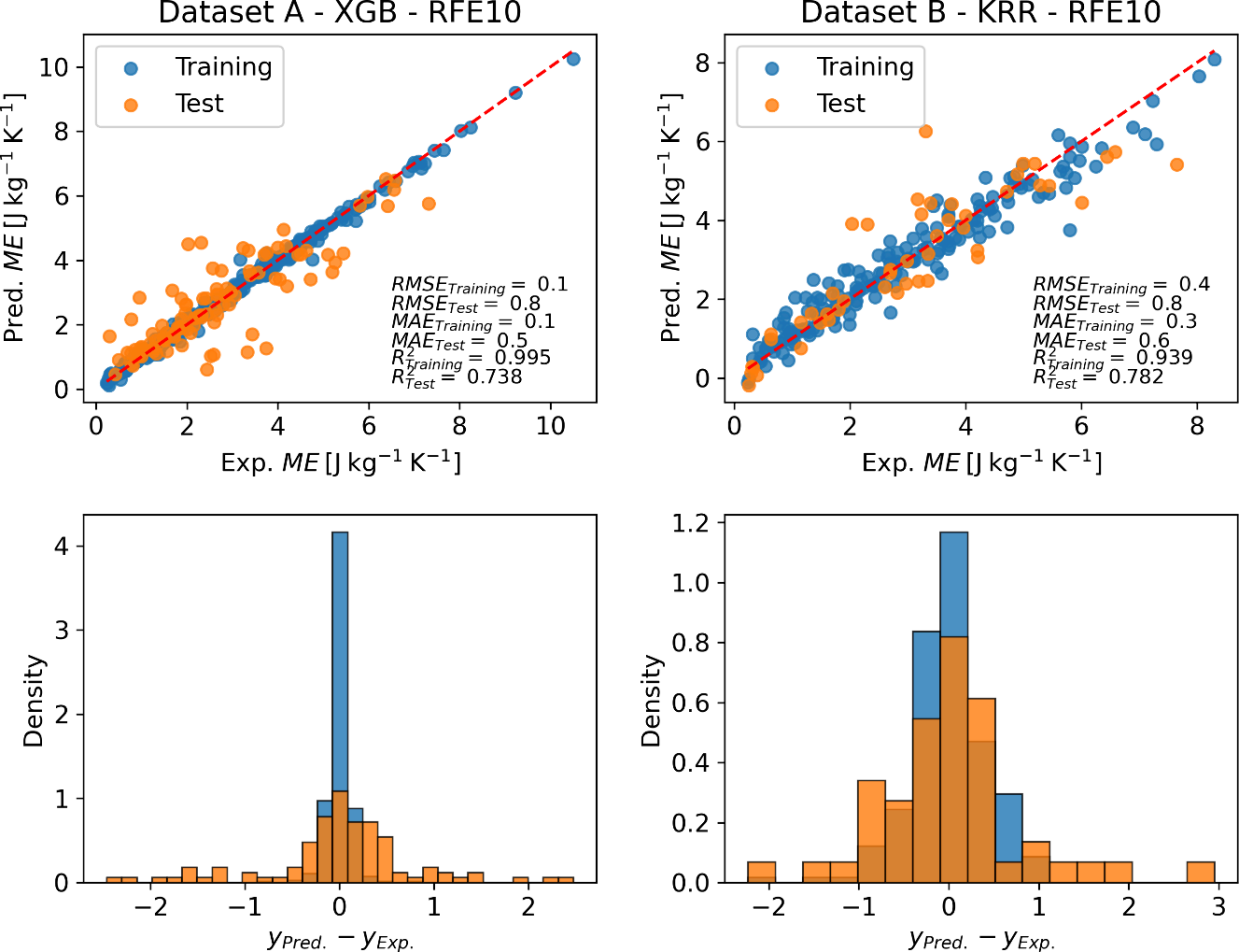
Parity plots of the best combination of ML models and feature set for experimental vs predicted ME of magnetocaloric perovskites for training (blue) and test (orange) data. Below each parity plot, the respective normalized histogram of the residuals is presented.

Figure 9 shows the best combination of the ML model and feature set for the ME prediction, the parity plots, and the performance metrics for Data Sets A and B. The figure also presents histograms of residuals, indicating the difference between the data collected and the data predicted for the models. Unlike the models for *T*_C_, the residual histograms of the models for both datasets exhibit the same mode for both test and training data, indicating consistency in the distribution of model errors across different datasets. However, there is a significant difference between the training and test data residuals and scores for dataset A, indicating an overfitting situation that could not be treated only by a cross-validation scheme and early stopping strategy, which points to relatively scarce data. Additionally, an examination of the validation curve (Figure S1) shows that, although there are differences in the determination coefficient between the training and test sets, the RMSE curves are continuously decreasing in both the training and validation sets.

According to the coefficient of determination metrics, our best model was KRR, using Dataset B and RFE10 as feature sets, resulting in RMSE of 0.8 J kg^–1^ K^–1^, MAE of 0.6 J kg^–1^ K^–1^ and *R*^2^ of 0.78. This is comparable with the XGBoost models built by Castro et al.^45^ and Court et al.,^44^ who both predict the peak entropy change of magnetocaloric materials utilizing the features generated by XenonPy and obtained a MAE of 1.8 J kg^–1^ K^–1^ (164 test data points) and MAE of 1.2 J kg^–1^ K^–1^ (along with *R*^2^ of 0.87 using 152 test data points), respectively. Additionally, Ucar et al.^22^ also built a RF regressor over a variety of magnetocaloric materials and obtained MAE of 2.26 J kg^–1^ K^–1^, RMSE of 3.23 J kg^–1^ K^–1^, and *R*^2^ equals 0.82. While keeping in mind the differences in the data quantity for each work, the results of our selection of the model and feature set seem promising, even when compared to the results with crystallite size, which also has a lower MAE (0.6 J kg^–1^ K^–1^). Although our MAE is better than the one reported by Court et al., our *R*^2^ is worse. These results must be verified with more data since the crystallite size is not commonly reported as described in the section dataset description, affecting the ML model’s accuracy.

In regards to the interpretation of the models, it was found that the magnetic field exerts the major influence on ME, and this isolated variable explains 26% and 33% of the variation of ME, in models for datasets A and B, respectively. Another important driven feature was the crystallite size which its effect is in average 10%. A detailed discussion is given in the Supporting Information under the section Interpretability of the ML models.

#### Relative Cooling Power

**Figure 10:**
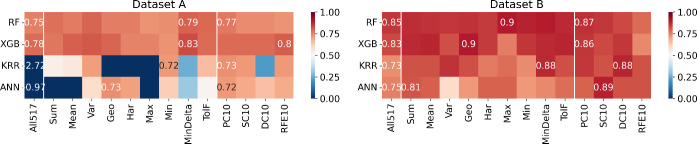
Heatmaps indicating the *R*^2^ in the prediction of relative cooling power (RCP), using different combinations of ML models (*y* labels) and feature sets (*x* labels). The squares with numbers in them represent better results among the feature sets. The performance using all compositional features (All517’) is always displayed.

**Figure 11:**
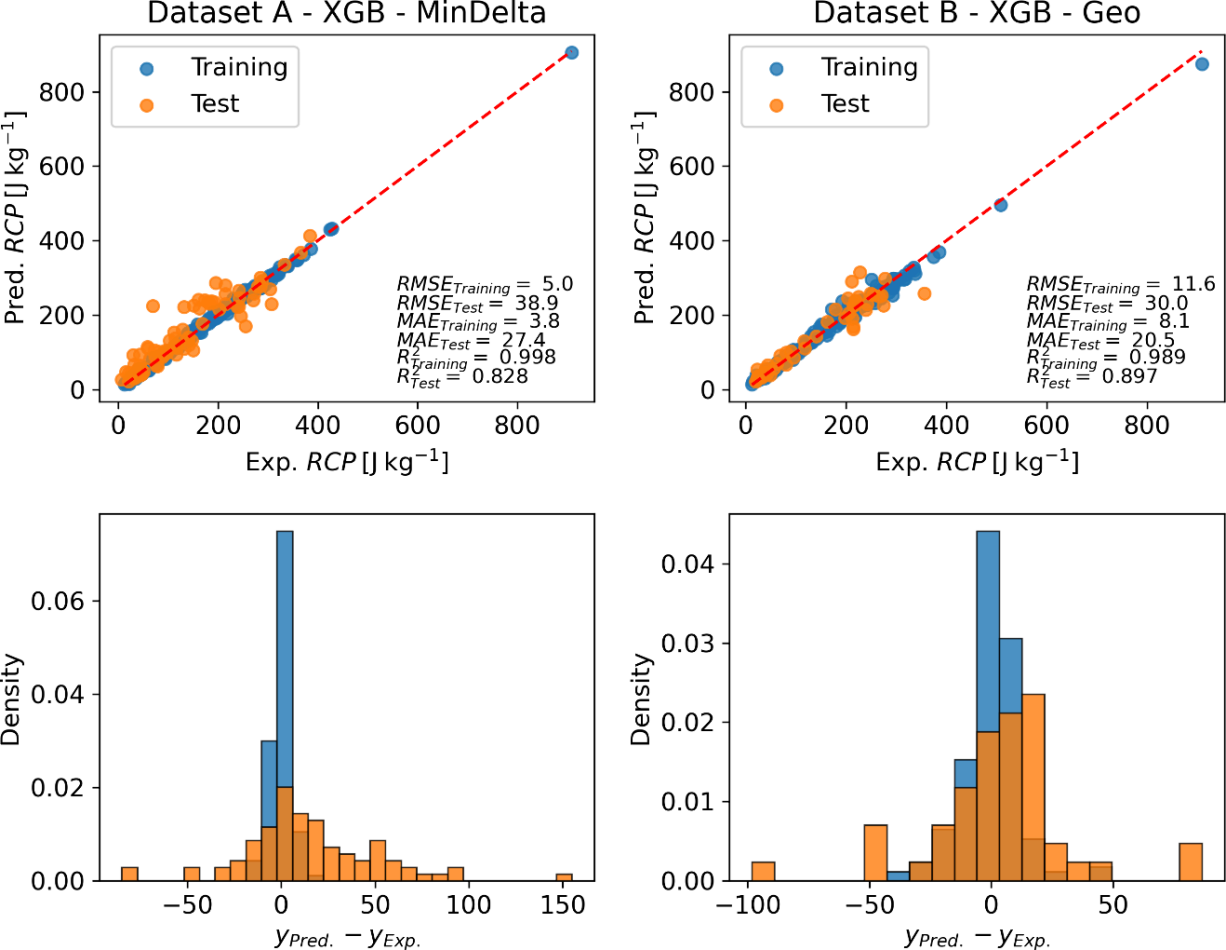
Parity plots of the best combination of ML models and feature set for experimental vs predicted *RCP* of magnetocaloric perovskites for training (blue) and test (orange) data. Below each parity plot, the respective normalized histogram of residuals is presented.

The outcomes for this particular property (Figure 10) reveal that the models trained with Dataset B exhibited superior performance compared to those trained with Dataset A. Notably, both KRR and ANN models demonstrated inferior performance relative to others when applied to Dataset A. Interestingly, across both datasets, no feature set stood out significantly among grouped and ranked features. This suggests the importance of feature selection across the various sets, indicating the complexity of the datasets and the challenges inherent in identifying dominant features for predictive modeling. The analysis of the histogram of residuals (presented in Figure 11) shows the same mode for training and test data in Dataset A, while for Dataset B, there is a tendency to overestimate. In both cases, a form of overfitting was observed. However, similar to the previous case, the validation curves (Figures S2 and S3 for dataset A and dataset B, respectively) show a simultaneous decrease in RMSE for both the training and validation data.

According to all the evaluation metrics, our best model was the XGBoost using Dataset B and Geometric Average as the best group of features, resulting in RMSE of 30 J kg^–1^, MAE of 20.5 J kg^–1^ and *R*^2^ of 0.89. This is comparable with the XGBoost model constructed by Court et al., which evidenced an MAE of 16.8 J kg^–1^ and *R*^2^ of 0.92 (88 test data points).

In regard to the feature influence in the RCP according to the interpretation of the ML models, it was found that the magnetic field exerts the highest influence in this property having an effect of 44% and 37% for the best ML model on dataset A and dataset B, respectively. On the other hand, crystallite size exerts 5% influence in the magnitude of this variable.

### Screening

In this section, we leverage the best ML models discussed in the previous sections to explore the chemical space of the perovskite magnetocaloric materials described in the method section. To this end, we employed the models trained with Dataset A. Due to its larger data size, these ensure greater robustness compared to models trained on Dataset B. Also, because the morphological parameter (crystallite size) does not remain a free parameter, we can focus only on chemical composition.

**Figure 12:**
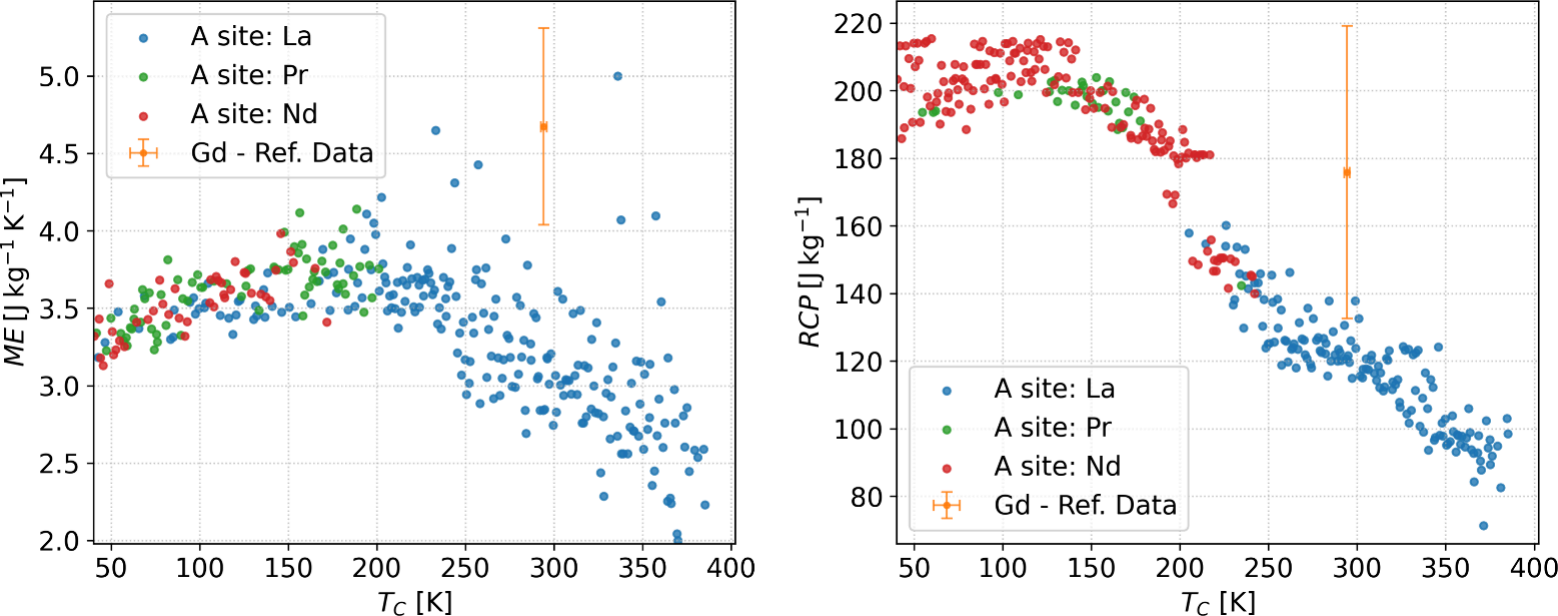
Contour of predicted *T*_C_ vs predicted ME (left) and predicted *T*_C_ vs predicted RCP (right) at 2 T for the chemical space of perovskite (i.e., only perovskites with the highest ME or RCP at each interval of 1 K of *T*_C_ were plotted). Colors refer to the principal element in the A-site.

To conduct this analysis, we employed the figure of merit schematized by Gottschall et al.,^46^ where *T*_C_ is located on the *x*-axis and RCP is an indicator of the intensity of the magnetocaloric effect on the *y*-axis. In this way, Figure 12 presents exclusively the composition with the highest predicted RCP (right) and ME (left), for each value of predicted *T*_C_ in the range from 40 to 400 K, increasing in 1 K increments. The compositions were categorized by the predominant chemical element present in the A-site (La, Nd and Pr). The magnetocaloric properties of Gadolinium (Gd), the benchmark magnetocaloric material for applications in ambient temperature,^47^ were also plotted for comparison. Gd magnetocaloric properties are plotted as the average of the available experimental data, and the error bar corresponds to the standard deviation. Details about the data collected for Gd is discussed in the Supporting Information.

The predicted ME values across the examined temperature range demonstrate significant scatter. At lower temperatures (*T*_C_ between 40 and 200 K), ME values start near 3.5 J/kgK, with compositions predominantly composed of manganites containing the three considered A-sites: Lanthanum (La), Praseodymium (Pr), or Neodymium (Nd). As the temperature increases beyond 200 K, the landscape shifts, and La-based manganites become increasingly dominant. Despite the broader scattering of ME values at higher temperatures, it is notable that none of the predicted compositions fall within Gd’s experimental error bars. However, a few compositions exhibit ME magnitudes comparable to that of Gd, albeit at slightly lower or higher *T*_C_.

The analysis of RCP reveals a similarly nuanced picture. For *T*_C_ values between 40 and 200 K, most compositions achieve an RCP of approximately 200 J/kg, with Nd-based manganites being the primary candidates, alongside a few Pr-based compositions. Moving to the intermediate temperature range of 200–250 K, the best-performing perovskites represent a mixture of La-, Pr-, and Nd-based manganites. However, RCP begins to decrease as the temperature increases. Above 250 K, La-based manganites exclusively emerge as the top candidates, although their performance declines further with increasing temperature. Interestingly, some La-based compositions near Gd’s *T*_C_ approach the lower limit of Gd’s error bars, with an RCP close to 130 J/kg. This proximity suggests that while these perovskites may not surpass Gd in performance, they could offer alternative pathways for optimization.

These findings underscore the varied influence of the A-site chemistry across temperature ranges. Nd-based manganites dominate at lower temperatures, while La-based compositions excel at higher temperatures. Although no perovskite in the considered chemical space matches Gd’s performance within its experimental error bars, certain La-based manganites approach its lower threshold. To further investigate this behavior and identify trends that could guide the exploration of new manganites for room temperature refrigeration, we now analyze the influence of doping at both the A-site and the B-site.

**Figure 13:**
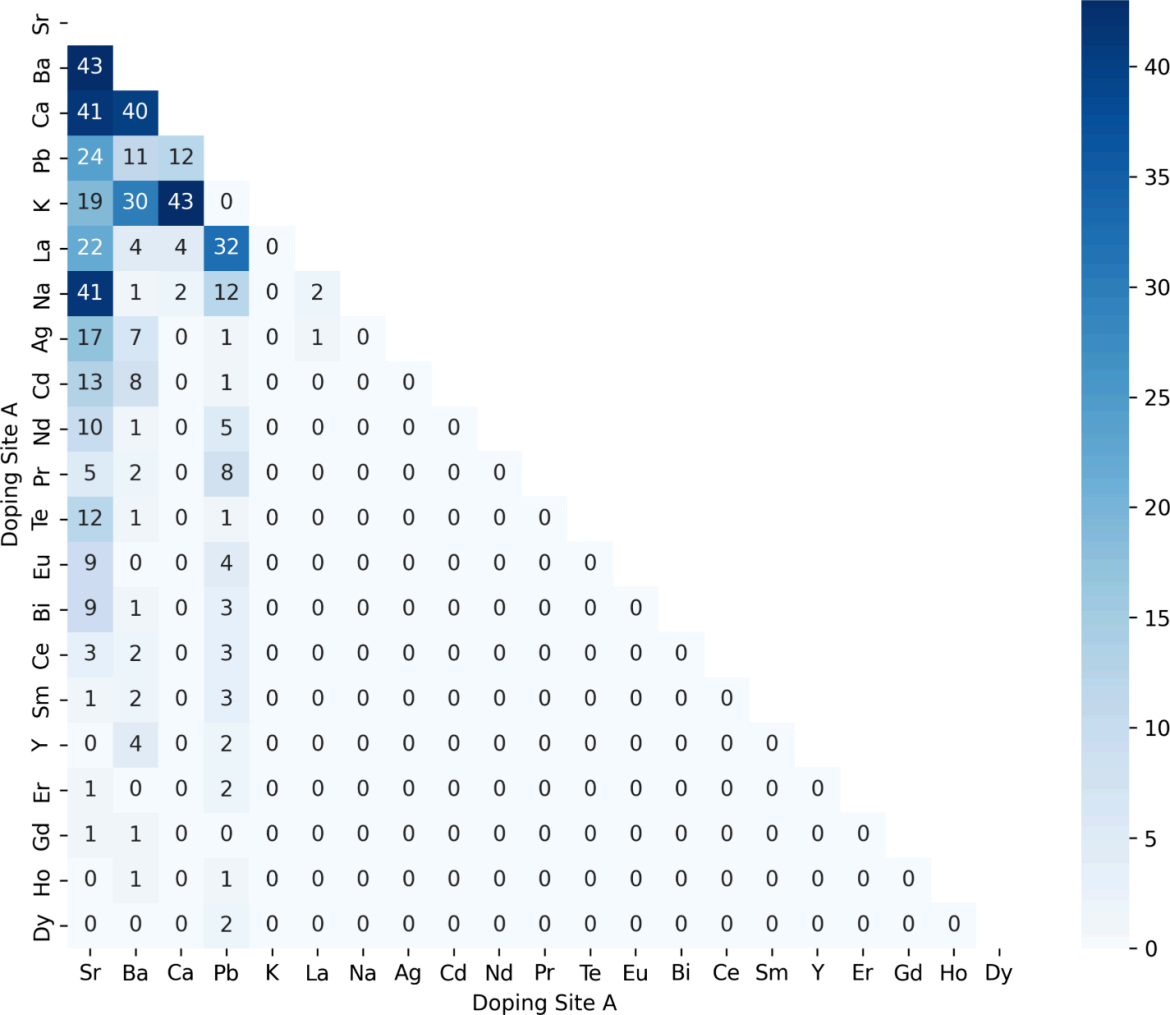
Co-occurrence matrix between the doping cations in the perovskite compositions that showed superior results for both ME and RCP and has a *T*_C_ approximately at room temperature, according to our best models for each property. The numbers in the legend refers to the quantity of perovskites compositions with both of the cations in the *x* and *y* axis.

**Figure 14:**
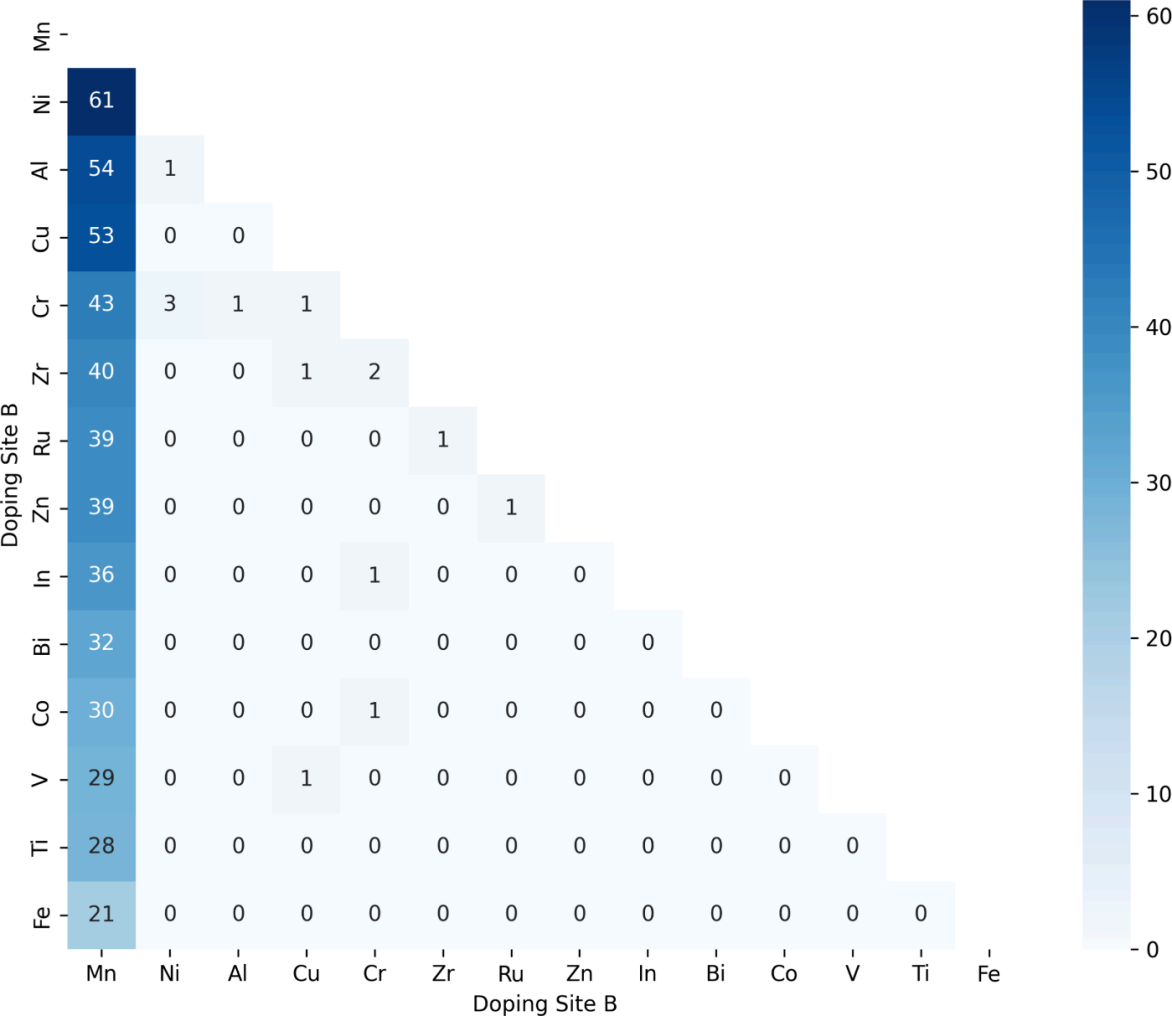
Co-occurrence matrix between the doping anions in the perovskite compositions that showed superior results for both ME and RCP and has a *T*_C_ approximately at room temperature, according to our best models for each property. The numbers in the legend refer to the quantity of perovskites compositions has both of the anions in the *x* and *y* axis.

The distribution of doping elements at sites A and B of perovskites in the top-performing compositions were analyzed through a co-occurrence matrix for each site (Figures 13 and 14). For this purpose, 1.2 million compositions were filtered to include only compositions with both ME and RCP values above the average (1.98 J kg^–1^ K^–1^ and 66.8 J kg^–1^ respectively), while ensuring that the *T*_C_ values fell within a range close to room temperature (288–318 K). This analysis revealed the frequency and co-occurrence patterns of different combinations of elements within the selected perovskites, which were exclusively La-based manganites i.e. La_0.6_A_a1_^(1)^A_a2_^(2)^Mn_0.8_B_b1_^(1)^B_b2_^(2)^O_3_. The results showed that strontium (Sr) is the element that occurs the most frequently as doping for site A, appearing in 290 of the 534 compositions that exhibited superior performance in all three properties. Additionally, combinations of Sr with barium (Ba), and calcium (Ca) were the most frequent codoping elements. Similarly, manganese (Mn) emerged as the most common element at site B, present in 520 of the 534 compositions as a doping element, with concentrations exceeding 0.8. Notably, combinations of Mn with nickel (Ni) and aluminum (Al) appeared more frequently between the top-performing perovskite compositions. These findings indicate that Sr and Mn are the most important doping elements to optimize the magneto-caloric properties of perovskite materials.

## Conclusions

This work involved developing and assessing 4 different machine-learning models by employing diverse feature sets to calculate the main magnetocaloric properties of perovskite materials. This approach can be applied to any group of magnetocaloric materials, with its primary limitations being data availability and quality. This study focuses on exploring a chemical subspace of approximately 1.2 million manganites for magnetocaloric applications. A database of the magnetocaloric properties of hundreds of perovskites was compiled by using data collected from the scientific literature. Data analysis revealed the importance of crystallite size as a morphological descriptor and its absence in more than half of the data collected. This led to our study being performed on the complete data, leaving crystallite size features apart and in the subdataset with this morphological parameter.

The features to train the model was derived by proportional combination of the atomic properties of the constituent elements of the perovskites, leading to 517 compositional descriptors. To evaluate this high dimensionality, different feature sets were proposed, based on the type of combination rule used to blend the atomic properties (grouped feature sets), and by taking the 10 most relevant features based on different ranking methods (ranked feature sets). After training the models of the different feature sets, the performance was assessed through the determination coefficient metric. The results present heterogeneous outcomes across the ML models and the dataset used. In general, it was found that the use of the whole set of 517 features effectively did not lead to the best results, confirming the necessity of feature selection. Furthermore, a comparison of this work models with those obtained in similar studies discloses alike or superior performance on each of the three magnetocaloric properties.

The predictive capabilities of the best models were leveraged to explore the magnetocaloric effect within the chemical subspace of manganites. As indicators of magnetocaloric intensity, both ME and RCP were employed. Trends were established for the main element in site A: (i) neodymium and praseodymium manganites are better suited for applications below 200 K, while (ii) lanthanum manganites perform better for higher temperature applications. For room temperature applications, additional trends were identified using a co-occurrence matrix. Specifically, strontium, barium, calcium, and lead were found to be the strongest candidates for enhancing La-based manganites for room temperature refrigeration. Regarding site B, nickel, aluminum, and copper emerged as the most frequent candidates for single doping alongside manganese. This analysis will provide a valuable guide for the experimental exploration of new combinations of doping elements for La-based manganites in the quest for economically feasible alternatives to gadolinium.

Finally, given the substantiated evidence of the relationship between crystallite size and all three magnetocaloric properties, we propose that future studies, particularly those employing DRX analysis, explicitly report crystallite size as a parameter. This recommendation aims to foster a more comprehensive understanding of the intricate connections between the crystallite size and magnetocaloric properties in perovskite materials.

## Data Availability

The Python scripts used for all stages of the methods, along with the data, are publicly available at https://github.com/luis-chemeng/MCML.

## Supporting Information Available

Combinational Formulas and Atomic properties; Hyperparameters of ML models; Validation Curves; Interpretability of the ML models; Gadolinium data (PDF)
