# Supplementary material for: Machine Learning Methodologies Applied to Magnetocaloric Perovskites Discovery
Source: J Chem Inf Model. 2025 Feb 6;65(4):1812–25. doi: 10.1021/acs.jcim.4c01944 (PMC11863371; doi:10.1021/acs.jcim.4c01944)
Supplement: Supplementary file 1 — ci4c01944_si_001.pdf [file ci4c01944_si_001.pdf]

Supplementary Material: Machine Learning Methodologies applied to Magnetocaloric Perovskites Discovery

Luis E. Castro-Anaya,† Eduardo Marese,† Jaime A. Lozano,‡ Guilherme F. Peixer,‡ Jader R. Barbosa Jr.,‡ and Sergio Yesid Gómez González*,†

†*Laboratory of Mass Transfer and Numerical Simulation of Chemical Systems, Department of Chemical Engineering and Food Engineering, Federal University of Santa Catarina (UFSC), Florianópolis, SC, 88040-900, Brazil*

‡*POLO - Research Laboratories for Emerging Technologies in Cooling and Thermophysics, Department of Mechanical Engineering, Federal University of Santa Catarina (UFSC), Florianópolis, SC, 88040-900, Brazil*

E-mail: sergio.gomez@ufsc.br

Combinational Formulas and Atomic properties

$$\text{Mean prop} = \frac{\sum w_i \cdot \text{prop}_i}{\sum w_i} \tag{S1}$$

$$\text{Sum prop} = \sum w_i \cdot \text{prop}_i \tag{S2}$$

$$\text{Var prop} = \frac{\sum w_i \cdot (\text{prop}_i - \text{Mean prop})^2}{\sum w_i} \tag{S3}$$

$$\text{Geo prop} = \left(\prod \text{prop}_i^{w_i}\right)^{\frac{1}{\sum w_i}} \tag{S4}$$

$$\text{Har prop} = \frac{\sum w_i}{\sum \frac{w_i}{\text{prop}_i}} \tag{S5}$$

$$\text{Max prop} = \max(\text{prop}_1, \text{prop}_2, \ldots, \text{prop}_N) \tag{S6}$$

$$\text{Min prop} = \min(\text{prop}_1, \text{prop}_2, \ldots, \text{prop}_N) \tag{S7}$$

$$\text{MinDelta prop} = \min(\text{Mean prop}_A - \text{prop}_O, \text{Mean prop}_B - \text{prop}_O) \tag{S8}$$

$$\text{TolF prop} = \frac{\text{Mean prop}_A + \text{prop}_O}{\text{Mean prop}_B + \text{prop}_O} \tag{S9}$$

where i refers to any chemical element at site A or site B, prop_i stands for the value of atomic property for the element i taking from XenonPy database and w_i is the concentration of element i within the chemical formula.

Table S1: Features Description

**Feature**	**Description**
applied_field	Field measurement for RCP and entropy models
atomic_number	Number of protons found in the nucleus of an atom
atomic_radius	Atomic radius
atomic_radius_rahm	Atomic radius by Rahm et al
atomic_volume	Atomic volume
atomic_weight	The mass of an atom
boiling_point	Boiling temperature

bulk_modulus	Bulk modulus
c6_gb	C_6 dispersion coefficient in a.u.
covalent_radius_cordero	Covalent radius by Cerdero et al
covalent_radius_pyykko	Single bond covalent radius by Pyykko et al
covalent_radius_pyykko_double	Double bond covalent radius by Pyykko et al
covalent_radius_pyykko_triple	Triple bond covalent radius by Pyykko et al
covalent_radius_slater	Covalent radius by Slater
density	Density at T = 295 K
dipole_polarizability	Dipole polarizability
electron_affinity	Electron affinity
electron_negativity	Tendency to attract a shared pair of electrons
en_allen	Allen's scale of electronegativity
en_ghosh	Ghosh's scale of electronegativity
en_pauling	Mulliken's scale of electronegativity
first$_{i}on_{e}n$	First ionisation energy
fusion_enthalpy	Fusion heat
gs_bandgap	DFT bandgap energy of T = 0 K ground state
gs_energy	DFT energy per atom of T = 0 K ground state
gs_est_bcc_latcnt	Estimated BCC lattice parameter
gs_est_fcc_latcnt	Estimated FCC lattice parameter
gs_mag_moment	DFT magnetic moment of T = 0 K ground state
gs_volume_per	DFT volume per atom of T = 0 K ground state
heat_capacity_mass	Mass specific heat capacity
heat_capacity_molar	Molar specific heat capacity
heat_of_formation	Heat of formation
hhi_p	Herfindahl-Hirschman Index production values
hhi_r	Herfindahl-Hirschman Index reserves values

icsd_volume	Atom volume in ICSD database
lattice_constant	Physical dimension of unit cells in a crystal lattice
melting_point	Melting point
mendeleev_number	Atom number in Mendeleev's periodic table
molar_volume	Molar volume
num_d_unfilled	Unfilled electrons in d shell
num_d_valence	Valence electrons in d shell
num_f_unfilled	Unfilled electrons in f shell
num_f_valence	Valence electrons in f shell
num_p_unfilled	Unfilled electrons in p shell
num_p_valence	Valence electrons in p shell
num_s_unfilled	Unfilled electrons in s shell
num_s_valence	Valence electrons in s shell
num_unfilled	Total unfilled electrons
num_valence	Total valence electrons
period	Period in the periodic table
poissons_ratio	Poisson's ratio
polarizability	Ability to form instantaneous dipoles
sound_velocity	Speed of sound
specific_heat	Specific heat at 20°C
thermal_conductivity	Thermal conductivity at 25°C
vdw_radius	Van der Waals radius
vdw_radius_alvarez	Van der Waals radius according to Alvarez
vdw_radius_mm3	Van der Waals radius from the MM3 FF
vdw_radius_uff	Van der Waals radius from the UFF

Hyperparameters of ML models

Table S2: KRR Hyperparameters

**Hyperparameter**	**Values**
alpha	[0.001, 0.003, 0.01, 0.03, 0.1, 0.3, 1, 3, 10]
kernel	['rbf', 'poly']
gamma	[0.001, 0.01, 0.1, 1, 10]

Table S3: ANN Hyperparameters

**Hyperparameter**	**Values**
Hidden layers	[4,), (8,), (12,), (4, 4), (12, 4), (12, 12), (4, 4, 4)]
Activation function	['relu', 'logistic']
alpha	[0.001, 0.003, 0.01, 0.03, 0.1, 0.3, 1, 3, 10]

Table S4: RF Hyperparameters

**Hyperparameter**	**Values**
n_estimators	[20, 40, 60, ..., 400]
max_depth	[2, 5, 10, None]
min_samples_split	[2, 5, 10]
min_samples_leaf	[1, 3, 5]
ccp_alpha	[0.01, 0.1, 0.5, 1.0]

Table S5: XGBoost Hyperparameters

**Hyperparameter**	**Values**
early_stopping_rounds	20
max n_estimators	500
max_depth	[2, 5, 10, None]
learning_rate	[0.01, 0.1, 0.2]
subsample	[0.6, 0.7, 0.8, 0.9]
colsample_bytree	[0.7, 0.8, 0.9]
reg_alpha	[0.01, 0.05, 0.1, 0.5, 1.0, 2.0, 4.0]

Validation Curves

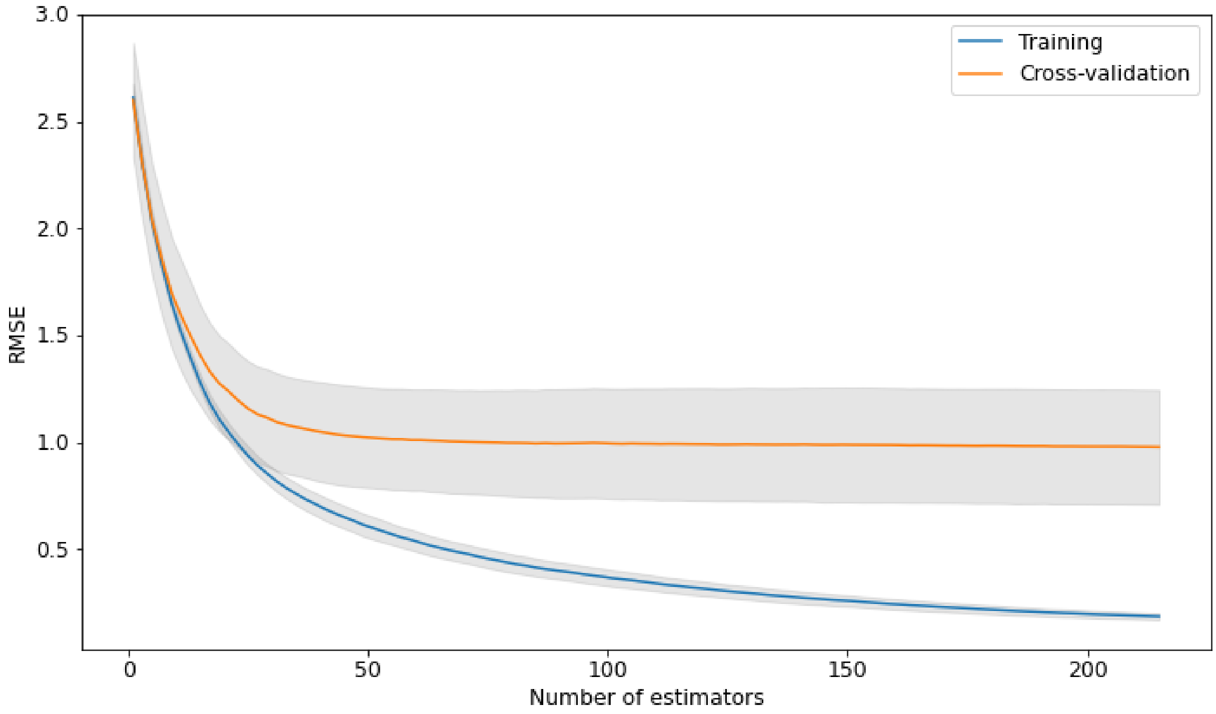

Figure S1: ME validation curve for the training and cross-validation sets using the XGB model in combination with the RFE10 feature set for dataset A. The gray area represents the standard deviation across the 5 cross-validation sets.

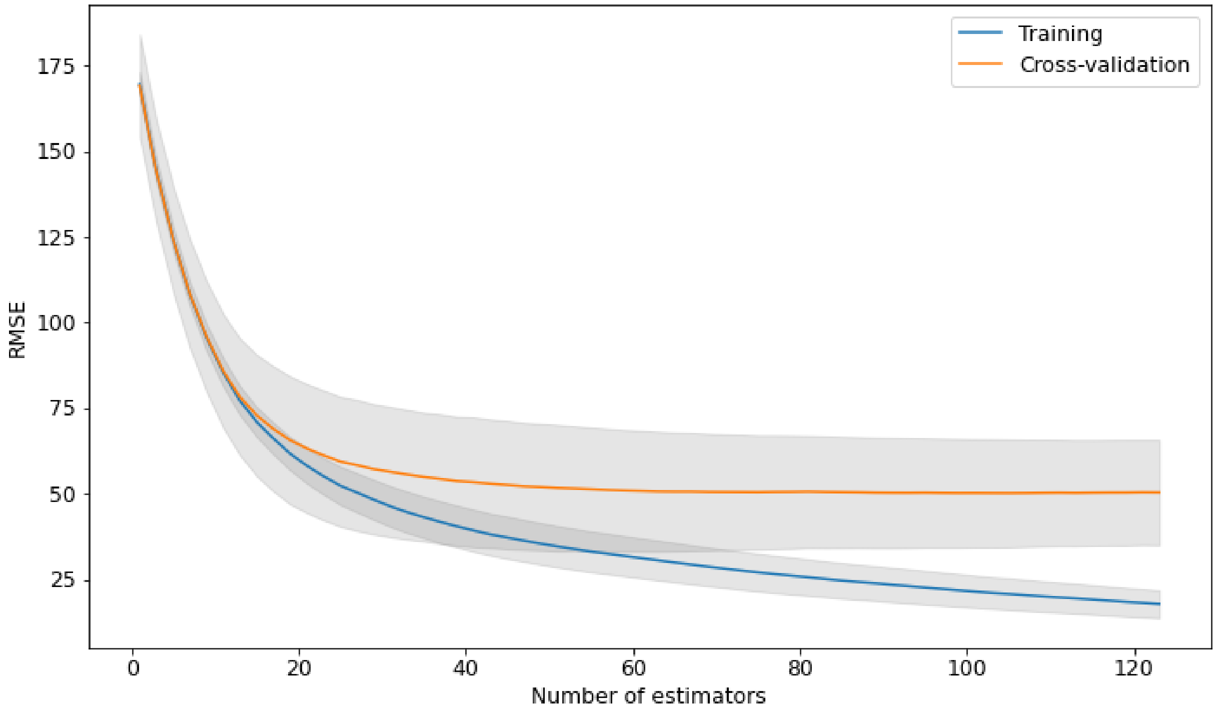

Figure S2: RCP validation curve for the training and cross-validation sets for XGB model in combination with MinDelta feature set for dataset A. The gray area represents the standard deviation across the 5 cross-validation sets.

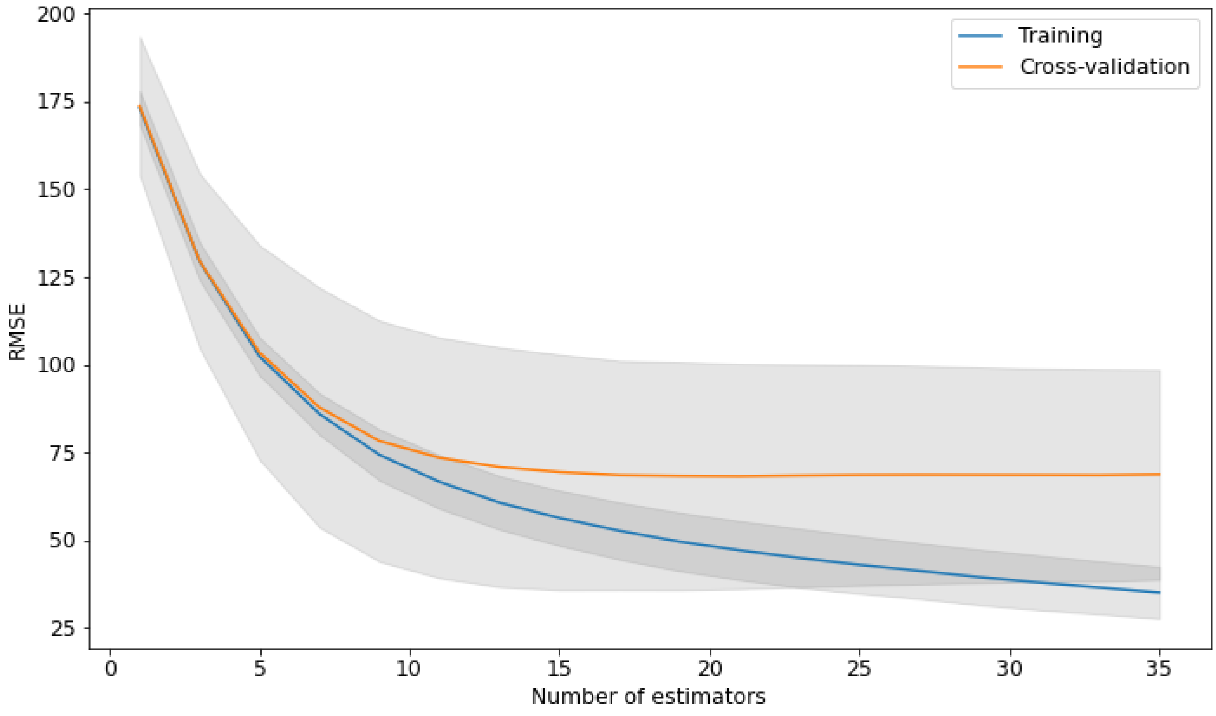

Figure S3: RCP validation curve for the training and cross-validation sets for XGB model in combination with Geo feature set for dataset B. The gray area represents the standard deviation across the 5 cross-validation sets.

Interpretability of the ML models

Machine learning (ML) models, in contrast to physical models, are often difficult to interpret due to their large number of parameters and inherently intricate architectures. As a result, the relationships between inputs and outputs are not easily interpretable.

To address this, we employ the Partial Dependence Plot (PDP) to analyze the average influence of an isolated feature on the output variable.[1] The PDP provides insight into the effect of one feature at a time. Recently, Greenwell et al.[2] proposed using the flatness of the PDP as a measure of feature importance. According to their approach, less flatness indicates higher influence on the output variable, while greater flatness corresponds to lower influence. They quantified this flatness by calculating the sample standard deviation of the PDP as a numerical measure of feature importance.

This analysis is useful, as it can be applied to any type of machine learning model. However, the picture becomes incomplete when dealing with correlated features, as it is unlikely for one feature to change without affecting others. This is particularly true for the compositional features considered in this study, where the atomic properties of the elements are not independent of one another. In this way the conclusions will be driven only around the independent features Magnetic Field (H) and Crystallite size (D). While for compositional features, some common trends between the models will be pointed out.

To normalize feature importance and make it unitless, we opted to use the coefficient of variation (i.e. the standard deviation divided by the mean) as the feature importance indicator. Figures S4-S6 present bar plots of the feature importance determined using this approach for the three magnetocaloric properties, respectively. Additionally, as crystallite size has proven to be a crucial parameter, Figures S7-S9 were created to illustrate its predicted behavior according to the ML models. In the following the main trends for each property are discussed:

The analysis for T_C (Figure S4) reveals the following: (i) In Dataset A, where the Variance

of the ground state magnetic moment was included as a feature, T_C exhibits the highest sensitivity to this feature, with an importance value of 0.3. (ii) In both datasets, the Variance of Allen's electronegativity was among the most relevant features, with importance values of 0.13 and 0.14 for Dataset A and Dataset B, respectively. A lookup at the PDP of this feature displays a negative trend, suggesting that a higher variance in the electronegativity of the elements in perovskites, on average, diminishes the T_C. (iii) In Dataset B, the morphology feature was the least significant variable, with a feature importance of 0.01. This suggests that the parameter has minimal impact on T_C, as illustrated for certain compositions in Figure S7.

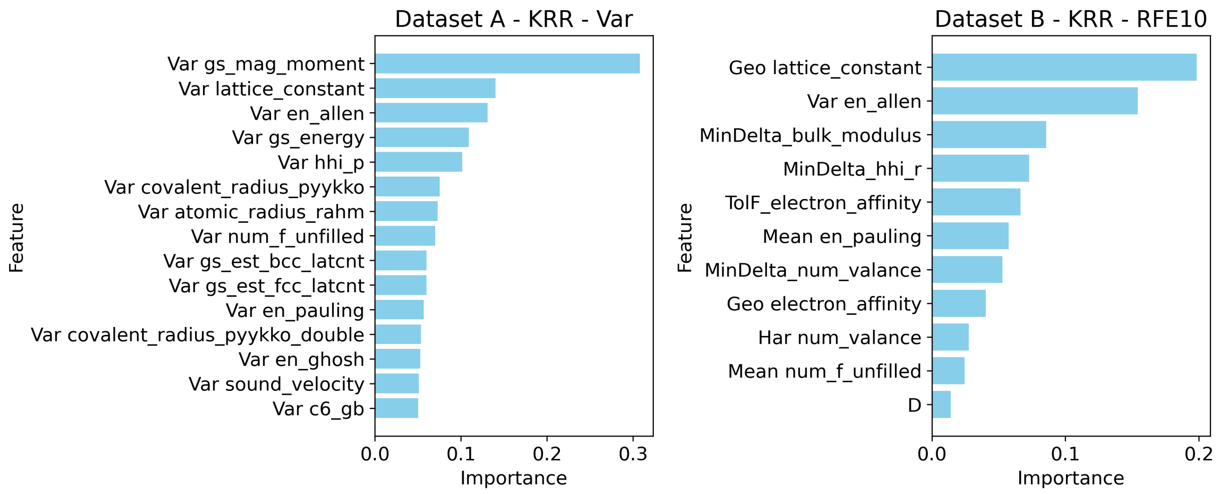

Figure S4: Feature importance coefficients for the optimal combination of ML models and feature sets in predicting T_C. The horizontal bars indicate the relative contribution of each feature to the prediction model. For clarity, a maximum of 15 features are displayed.

Regarding ME, the analysis reveals the following: (i) The magnetic field (H) in models for Dataset A and Dataset B had the greatest effect on ME, with importance values of 0.26 and 0.33, respectively. (ii) In the model for Dataset B, the crystallite size (D) exhibited an importance of 0.1. This indicates that ME is sensitive to the crystallite size, as shown in Figure S8. (iii) The remaining features did not display apparent similarities between the datasets. For instance, the second most important feature in Dataset A, with an importance value of 0.21, had a much lower importance of 0.05 in Dataset B.

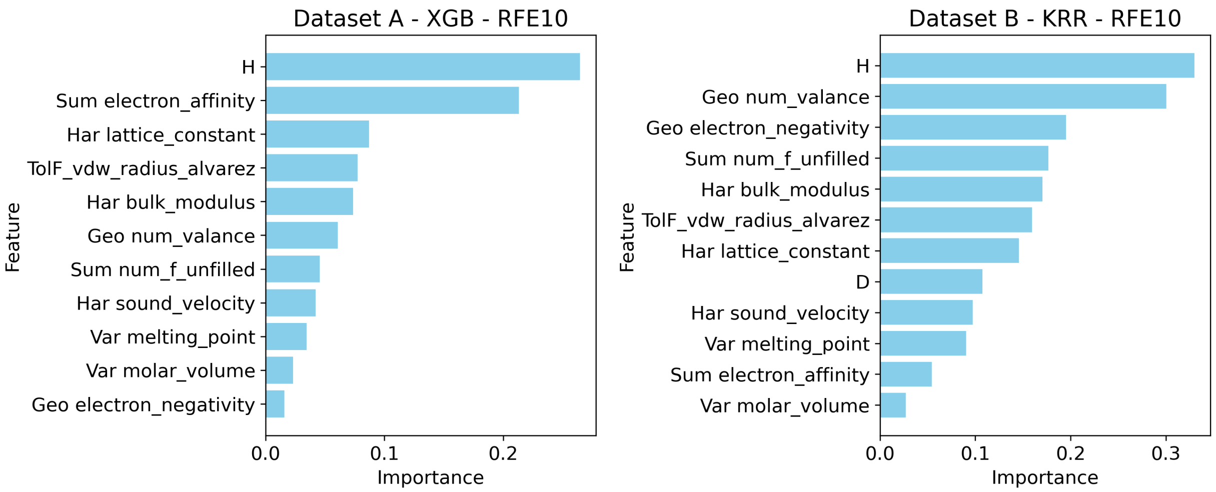

Figure S5: eature importance coefficients for the optimal combination of ML models and feature sets in predicting ME. The horizontal bars represent the relative contribution of each feature to the prediction model. For clarity, a maximum of 15 features are displayed.

For RCP models, the analysis shows the following: (i) Similar to the ME analysis, the magnetic field (H) was the dominant feature, with importance values of 0.44 and 0.37 for Dataset A and Dataset B, respectively. (ii) The importance of the crystallite size as a feature was 0.05, indicating an intermediate relevance compared to its effect on T_C and ME. This can be observed in Figure S9. (iii) For compositional features, no apparent parallels were found between the two datasets.

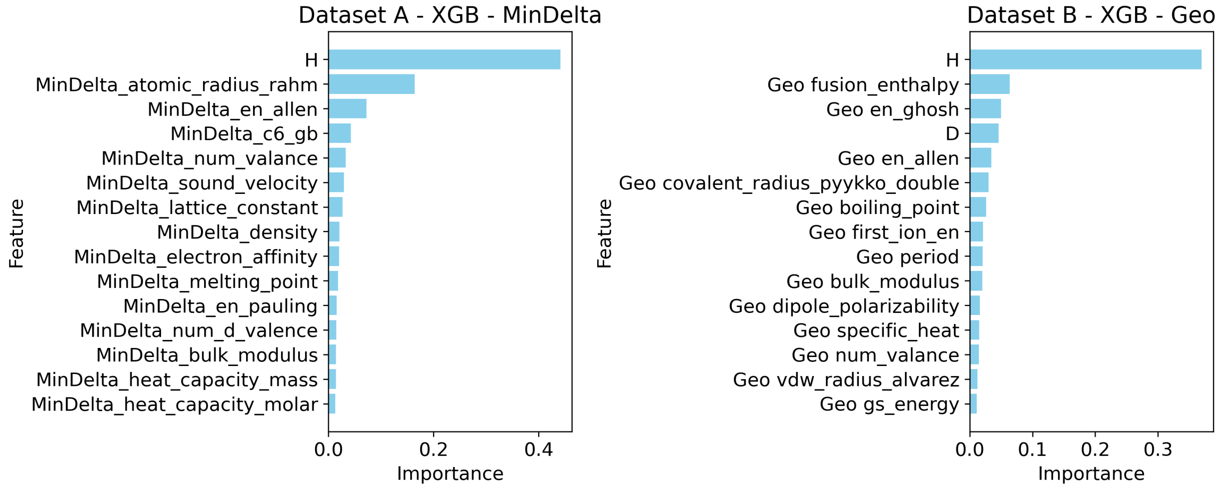

Figure S6: Feature importance coefficients for the optimal combination of ML models and feature sets in predicting RCP. The horizontal bars represent the relative contribution of each feature to the prediction model. For clarity, a maximum of 15 features are displayed.

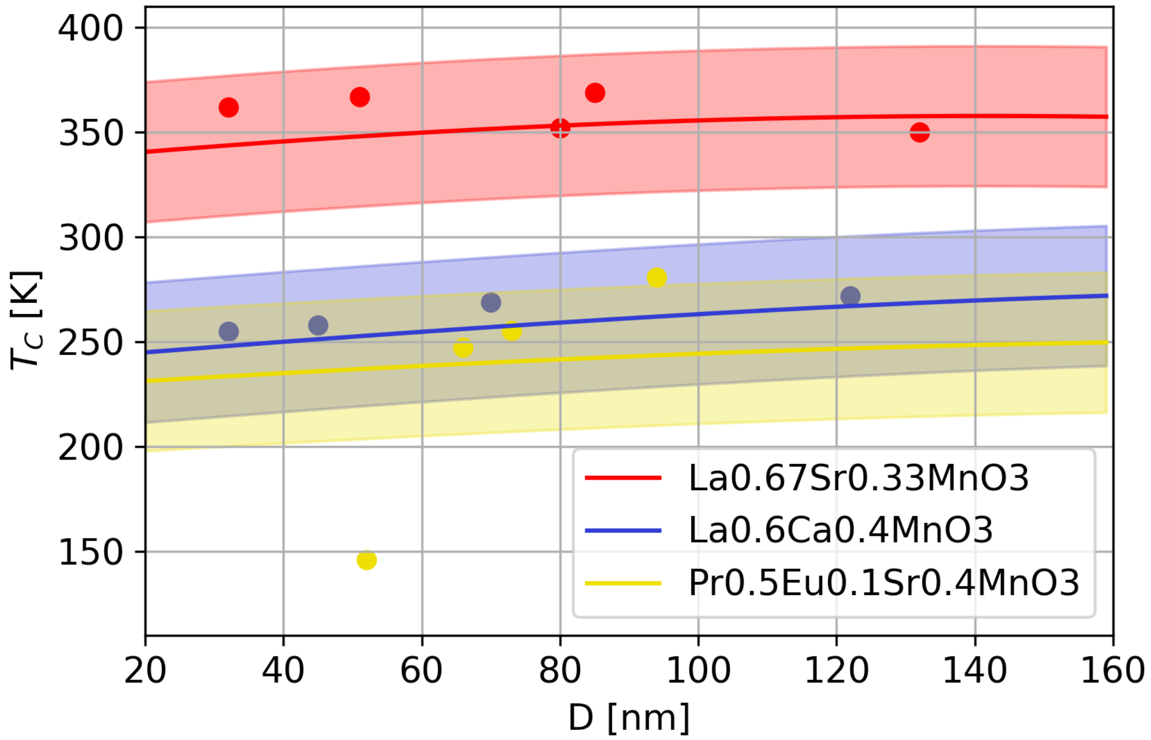

Figure S7: ML predictions of the effect of crystallite size on TC (solid lines) for three perovskites with the highest amount of experimental data (points). The shaded area represents uncertainty, reported as the RMSE of the test set.

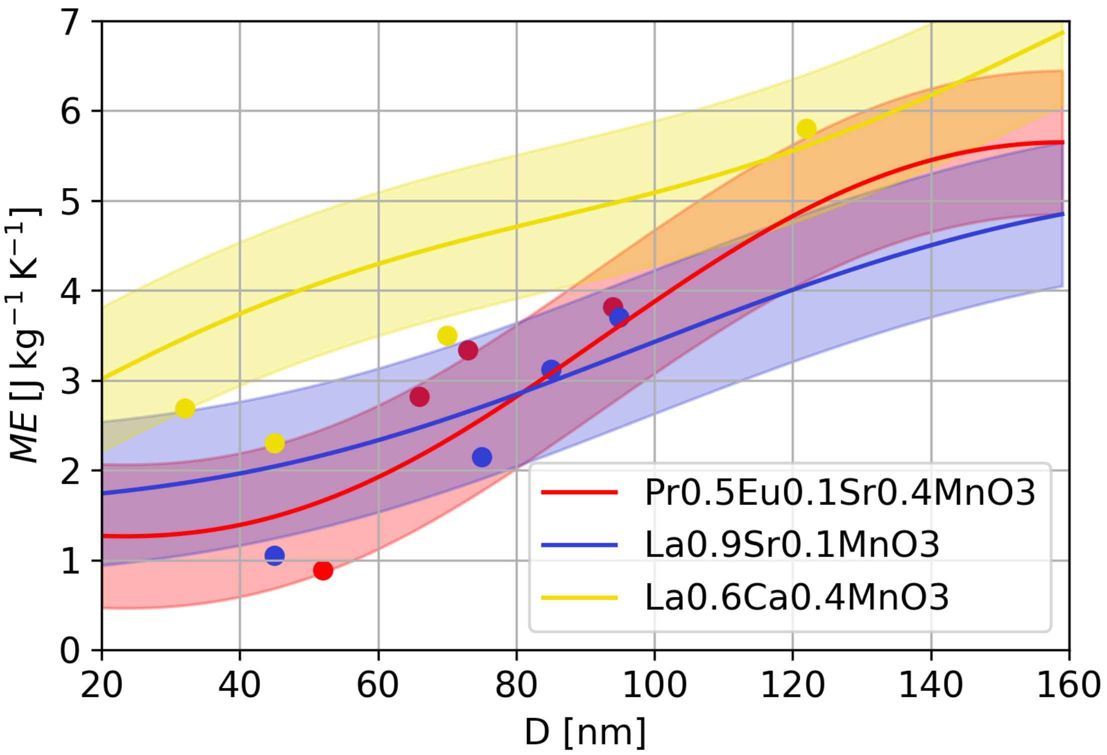

Figure S8: ML predictions of the effect of crystallite size on ME (solid lines) for three perovskites with the highest amount of experimental data (points). The shaded area represents uncertainty, reported as the RMSE of the test set.

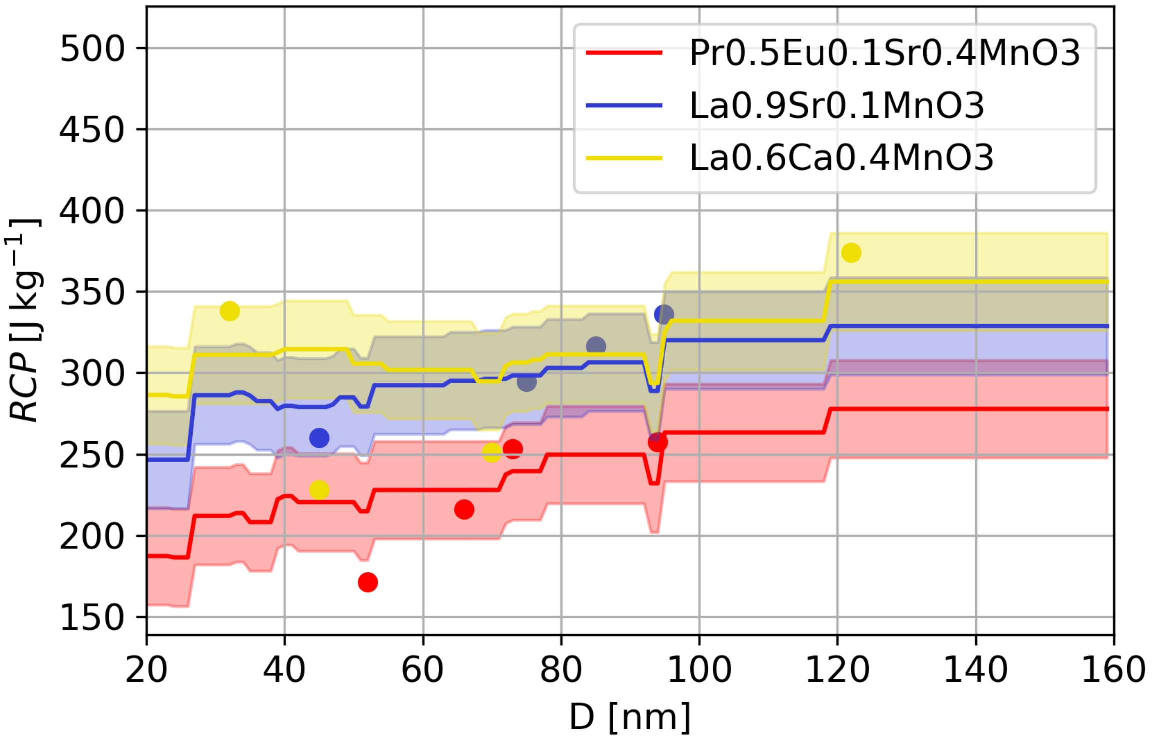

Figure S9: ML predictions of the effect of crystallite size on RCP (solid lines) for three perovskites with the highest amount of experimental data (points). The shaded area represents uncertainty, reported as the RMSE of the test set.

Gadolinium data

Surprisingly, we found few experimental data on the magnetocaloric properties of Gadolinium. The three magnetocaloric properties studied in this work were obtained from entropy curves. Figure S10 shows the only available collection of entropy curves for Gadolinium at a magnetic field of 2 T. As can be seen, the curves exhibit several differences in magnitude, likely related to the effect of crystallite size. This phenomenon was evidenced in the work of Zeng et al.,[3] who studied three samples of Gadolinium with different treatments and, consequently, different crystallite sizes. They found an increase in the magnitude of the magnetic entropy effect with an increase in crystallite size, as well as an increase in Curie temperature.

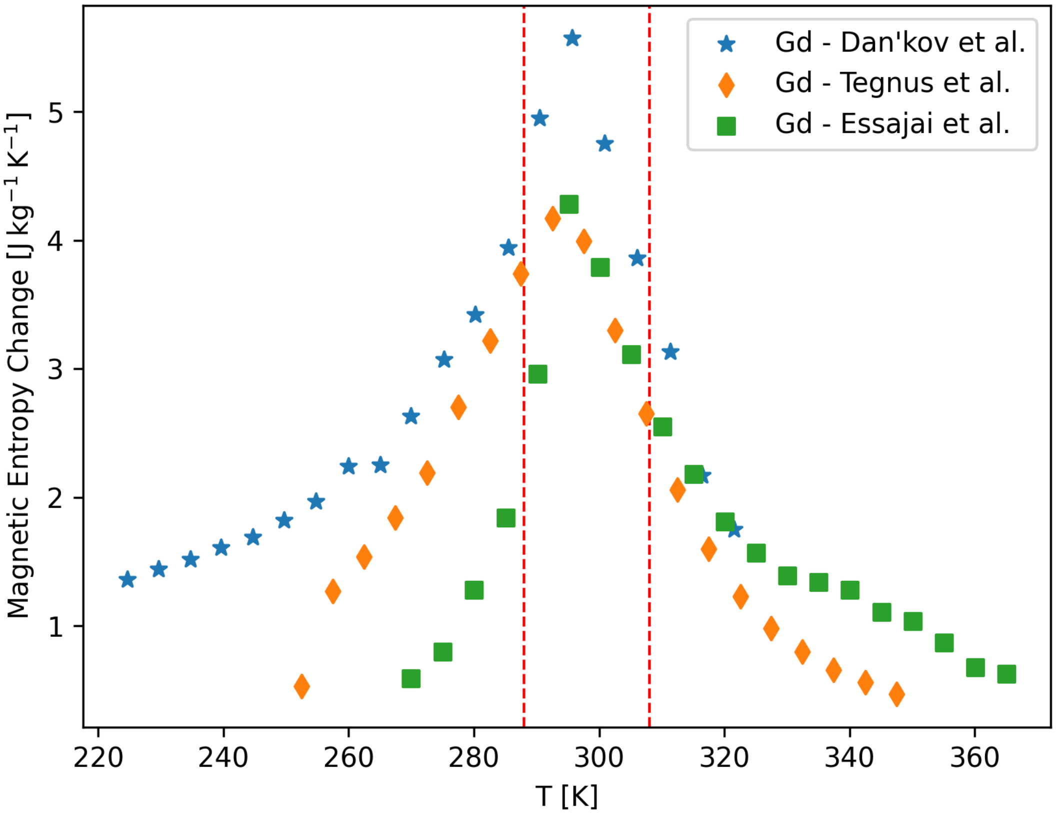

Figure S10: Curve of Magnetic Entropy Change over Temperature at 2 T for Gadolinium and two different perovskites compositions. The red dotted lines correspond to the interval of temperature from 288 K to 308 K.

References

(1) Friedman, J. H. Greedy function approximation: a gradient boosting machine. *Annals of statistics* **2001**, 1189–1232.

(2) Greenwell, B. M.; Boehmke, B. C.; McCarthy, A. J. A simple and effective model-based variable importance measure. *arXiv preprint arXiv:1805.04755* **2018**,

(3) Zeng, H.; Zhang, J.; Kuang, C.; Yue, M. Magnetic entropy change in bulk nanocrystalline Gd metals. *Applied Nanoscience* **2011**, *1*, 51–57.
